# Directing Neutrophil Fate via Sensory–Immune Interactions Accelerates Diabetic Bone Healing

**DOI:** 10.34133/research.1320

**Published:** 2026-06-24

**Authors:** Xuanyu Qi, Yijia Huang, Yuran Li, Jun Chen, Mingliang Zhou, Li Mei, Zeqian Xu, Sihan Lin, Xinquan Jiang

**Affiliations:** ^1^ Shanghai Stomatological Hospital, Fudan University, Shanghai 201102, China.; ^2^ Department of Prosthodontics, Shanghai Ninth People’s Hospital, Shanghai Jiao Tong University School of Medicine, Shanghai, 200011, China.; ^3^ College of Stomatology, Shanghai Jiao Tong University, Shanghai 200125, China.; ^4^ Department of Oral Sciences, Faculty of Dentistry, University of Otago, Dunedin, New Zealand.

## Abstract

The intractability of diabetic bone defects mainly results from derailed inflammation. While peripheral neuropathy is a common comorbidity, whether sensory dysfunction contributes to uncontrolled inflammation in diabetes is poorly understood. Here, within diabetic bone defects, we show that diminished sensory innervation is coupled with disrupted immune dynamics, characterized by both delayed neutrophil chemotaxis and abnormal neutrophil retention that resulted from impaired macrophage efferocytosis. Therefore, we design a chocolate chip cookie-like scaffold, in which the surface-embedded microspheres function as “chips” enabling burst interleukin-8 (IL-8) release, while the surrounding matrix provides sustained nerve growth factor release from silk fibroin matrix. Timely neutrophil chemotaxis induced by IL-8 triggers bone healing via stem cell recruitment, which is reinforced by sensory innervation by inducing neutrophil N2 polarization. Notably, macrophages preferentially established intimate physical proximity to outgrowing neurites to form a synapse-like structure, where they restore efferocytosis driven by neuronal Galectin-3. Moreover, spatiotemporally regulating neuroimmune circuit enhances mandibular bone regeneration in diabetic rats, highlighting the therapeutic potential of neuroimmune interaction in programming diabetic inflammation resolution.

## Introduction

Diabetes mellitus (DM) is posing tremendous burden on public health at an unprecedented level. According to the International Diabetes Federation, in 2024, 589 million adults globally suffer from diabetes in their lifetime. Chronic nonhealing wounds, a major complication of diabetes, affect nearly 1 in 10 people worldwide, one of the most debilitating being impaired bone healing [1–5]. A myriad of factors can prolong the recovery of bone defects in DM up to 87% [1], among which pronounced immune imbalance plays the most critical role [6,7]. The physiological bone healing process comprises sequential, overlapping phases of inflammatory, bone regrowth, and remodeling [1]. Under hyperglycemia, however, altered fate of immune cells, especially neutrophils, usually results in stagnation in the inflammatory phase rather than progression to the subsequent repair phase [8–11]. Relevant treatment strategies remain limited, due in part to an incomplete understanding of the inflammation-to-regeneration transition mechanisms.

Spatiotemporal interplay between neutrophils and macrophages at inflammatory phase is considered an “engine” that facilitates progression of healing [9,12]. Neutrophils are the first wave of immune cells that migrate to injured site and combat infectious threat. Growing evidence has highlighted the alternative functions of neutrophils in tissue healing parallel to their antibacterial functions, including direct or indirect effects on angiogenesis and cell proliferation [13,14]. The second wave of immune cells dominant at the injured sites are macrophages differentiated from recruited circulating monocytes. Macrophages engulf short-lived neutrophils by efferocytosis to turn off the damage-causing pro-inflammatory behaviors of neutrophils. Such spatiotemporal inflammation pattern is tightly orchestrated for proper healing to occur. In DM, conversely, a multitude of dysregulated neutrophils and macrophages in the stalled inflammation phase severely hinders wound healing. Abnormal recruitment of neutrophils and aberrant neutrophil extracellular trap (NET) activation underscore their role in nonhealing diabetic wounds [10,15]. However, how diabetes induces neutrophil abnormal retention remains poorly understood. The removal of apoptotic cells by macrophage efferocytosis is a critical step in immune homeostasis of bone [16], whereas under constant high glucose levels, defective efferocytosis in macrophages has been recognized to cause unresolved inflammation in pancreatic islet [17,18], adipose tissue [19], and skeletal muscle [20]. Therefore, normalizing the spatiotemporal pattern of neutrophil infiltration and macrophage efferocytosis emerges as a promising strategy to expedite the healing process of diabetic bone defects.

Nearly 50% of diabetic patients suffer from peripheral neuropathy (PN), characterized by axonal degeneration, loss of distal fibers, and segmental demyelination [21]. Of note, aggregate clinical evidence indicates the strong correlation between the presence of PN and substantial elevated risk of bone fracture in diabetic patients, suggesting the possibility that PN may directly contribute to diabetic bone disease [20,22,23]. Bone is highly innervated, and interplay between peripheral nerve and bone has become a hot spot of recent research efforts. For instance, sensory nerves now have been proved to play an indispensable role in the bone microenvironment during development and repair [24]. However, theoretical basis linking PN to bone health is still hidden in the corner. Until recently, the regulation of sensory nerves in tissue homeostasis by immunoregulation—either activating or suppressing immune cell function—has gradually been revealed [25]. These findings give us the hint that diabetic neuropathy may influence the inflammation progression to disrupt the bone healing.

Another important question is how sensory nerves regulate immune responses. Previous studies mainly focused on the mediators involved in the neural immunomodulation, overlooking the way by which this regulation precisely occurs [26,27]. “Neuro-immune synapse” is a specialized contact zone between immune cells and neurites for the functional and molecular interconnections [28,29]. Such structure was initially identified between sympathetic neurons and spleen T cells [30], and similar functional structures were later discovered between neurites and mast cells [31,32]. More recently, such structures have also been found to serve as the structural basis of neuronal regulation on tumor progression [33]. The close cell–cell interactions ensure efficient and targeted information relay for control of immune homeostasis. Nevertheless, whether similar structures are involved in neuroimmune regulation within bone microenvironment has rarely been reported.

In this study, we aim to explore whether sensory innervation can orchestrate the immune dynamics, thereby facilitating bone healing in DM. Based on the transcriptomes of bone tissue from mice and patients, as well as in vivo animal experiments, we depicted the spatiotemporal inflammation pattern at diabetic bone defect sites, which was marked by a lagged but persistent neutrophilic influx and impaired efferocytosis in macrophages. Inspired by this, we designed a novel, biomimetic strategy for reprogramming the inflammatory bone environment through biomimetic spatiotemporal coordination of neutrophil chemotaxis and sensory innervation, using a digital light processing (DLP)-based “chocolate chip cookie-like” (CCC) scaffold. Specifically, biomimetic spatiotemporal delivery of interleukin-8 (IL-8) and nerve growth factor (NGF) from the scaffold incited acute neutrophil response and sustained sensory ingrowth, thereby accelerating diabetic bone healing. Mechanistically, IL-8, as well as sensory nerves, strengthened N2 polarization of the neutrophil to rejuvenated stem cell recruitment. Furthermore, sensory neuron–macrophage synapse-like connection ignited efferocytosis to dampen excessive inflammation via Galectin-3 (Gal-3)–Mer tyrosine kinase (MerTk) axis. This work sheds light on the underappreciated role of damaged early neutrophil chemotaxis and sensory nerves in orchestrating timely inflammation resolution and offers an alternative option for the treatment of diabetic bone diseases.

## Results

### Diminished sensory innervation was coupled with disrupted immune flow in diabetic bone defects

**Fig. 1.**
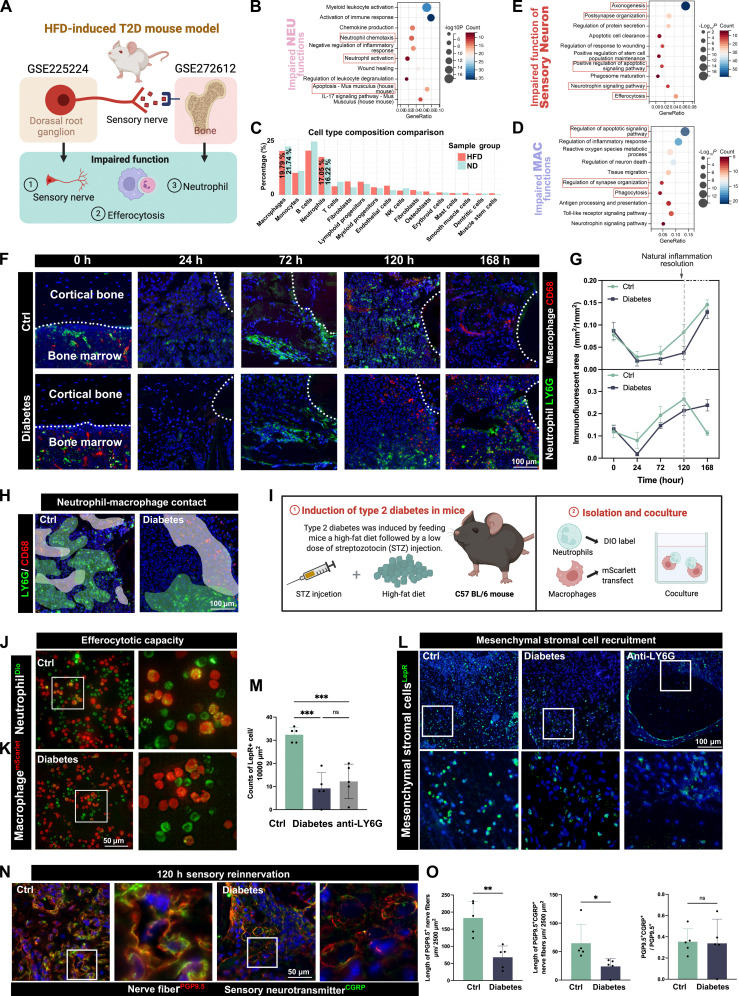
Diminished sensory innervation was coupled with disrupted immune flow in diabetic bone defects. (A) Schematic representation of transcriptome data analysis of the RNA-seq in GEO database. (B) GO/KEGG enrichment analysis of the DEGs in neutrophils (NEU) based on scRNA-seq data (GSE272612). (C) Comparison of cell type percentage in periosteal cells. (D) GO/KEGG enrichment analysis of the DEGs in macrophages (MAC). (E) GO/KEGG enrichment analysis of the DEGs in DRG based on bulk RNA-seq data (GSE225224). (F) Representative immunofluorescence images and (G) the semiquantitative analysis showing innate immune cell infiltration at serial time points (0 to 168 h) during bone healing in control and diabetic mice, stained for CD68 (red, macrophages) and LY6G (green, neutrophils). White dashed lines indicate the boundary of the cortical bone. Scale bars, 100 μm. (H) Neutrophil–macrophage contact analysis. Green area represents neutrophil infiltration, and white area represents macrophage infiltration. (I) Schematic representation and (J and K) representative image assessment of efferocytosis observed in coculture of mScarlet-labeled macrophages and DIO-labeled neutrophils, following 72-h incubation in control medium (Ctrl) and DCM (Diabetes). Scale bar, 50 μm. (L) Representative images and (M) semiquantitative assessment of MSC stained for LeptinR. Scale bars, 100 μm. (N) Representative images and (O) semiquantitative assessment of sensory nerve stained for CGRP and PGP9.5. Scale bars, 50 μm. Data are mean ± SEM analyzed by one-way ANOVA and post hoc multiple comparisons; ns indicates no statistically significant difference; *P < 0.05, **P < 0.01, ***P < 0.001, ****P < 0.0001.

To investigate the potential correlation between PN and inflammation outburst in diabetic bone, we reanalyzed published gene transcriptional profiles from mouse long-bone cells [single-cell RNA-sequencing (scRNA-seq), GSE272612] [34] and dorsal root ganglion (DRG) neurons (bulk RNA-seq, GSE225224) [35] (Fig. 1A and Fig. S1A to C). Although diabetes has long been recognized as an inflammatory disease [36], Gene Ontology (GO)/Kyoto Encyclopedia of Genes and Genomes (KEGG) enrichment analysis revealed impaired neutrophil chemotaxis and activation in high-fat diet (HFD)–induced diabetic mice compared with the normal diet (ND) group (Fig. 1B). However, HFD led to an increase in neutrophils (from 16.22% to 17.05%) (Fig. 1C), suggesting a dual impairment of diabetic neutrophils, with abnormalities in both activation and resolution. Suppressed neutrophil apoptosis, together with decreased macrophages (from 21.74% to 19.79%) and impaired macrophage phagocytosis, indicates that such neutrophil retention might result from dysfunctional macrophage efferocytosis of neutrophils (Fig. 1, B and D). Notably, the concurrent down-regulation of synapse-related activity in macrophages and sensory neurons under hyperglycemia suggested a potential neuro–immune interaction. The impaired axonogenesis of sensory neurons, together with their reduced regulation of apoptosis–efferocytosis, further supports a link between neural dysfunction and defective inflammatory resolution (Fig. 1E).

To validate the RNA-seq findings, we established a diabetic mouse model of femoral bone defect and monitored neutrophil (LY6G^+^) and macrophage (CD68^+^) infiltration during repair (Fig. 1F). Murine type II diabetes induction and femoral bone defect model construction were performed as previously described [37,38]. Following injury, neutrophils rapidly infiltrated the defect within 24 to 72 h, peaking around 120 h before sharply declining to baseline levels by 168 h. This decline coincided with increased macrophage accumulation, suggesting the role of macrophages in resolving neutrophil-driven inflammation (Fig. 1F, upper, and G). In diabetic mice, the early neutrophil influx was nearly absent within the first 72 h post-injury and gradually progressed into a state of persistent neutrophilic overflow (Fig. 1F, lower, and G). A clear spatial dissociation between macrophages and neutrophils was observed in the diabetic group compared with controls, despite a comparable late-phase increase in macrophage numbers. This finding indicated a potential impairment of macrophage-mediated efferocytosis in clearing neutrophils under diabetic conditions (Fig. 1H). We further cocultured neutrophils and macrophages isolated from DM bone tissue under hyperglycemia, with neutrophil membranes labeled by 3,3′-dioctadecyloxacarbocyanine perchlorate (DIO) and macrophages expressing mScarlet (Fig. 1I). Fluorescence imaging revealed a reduced red–green overlap in diabetic cells, indicating markedly impaired macrophage efferocytotic capacity (Fig. 1J and K).

We wondered whether delayed acute immune response hindered bone repair in DM. As the earliest responders to injury, neutrophils have long been overlooked for their essential contribution to tissue repair. Recently, evidence has indicated the role of neutrophils in initiating regeneration by recruiting mesenchymal stromal cells (MSCs) [39,40]. Consistently, either neutrophil depletion or DM induction exhibited diminished recruitment of stem cells to the injured sites at the late stage of the inflammation phase (120 h), underscoring the essential role of timely neutrophil infiltration in initiating regenerative cascade (Fig. 1L and M). Finally, consistent with the transcriptome sequencing, CGRP^+^/PGP9.5^+^ nerve innervation was impaired in diabetic bone defects (Fig. 1N and O). Our previous study demonstrated that sensory nerves terminated inflammation and initiated regeneration during bone repair [38]. Moreover, sensory nerves have been shown to modulate immune responses across multiple tissues [26,27,41,42]. These observations motivated us to explore the role of sensory reinnervation in restoring the disrupted spatiotemporal pattern of inflammation in DM.

### Timely reviving neutrophil chemotaxis and sensory innervation for promoting MSC recruitment

**Fig. 2.**
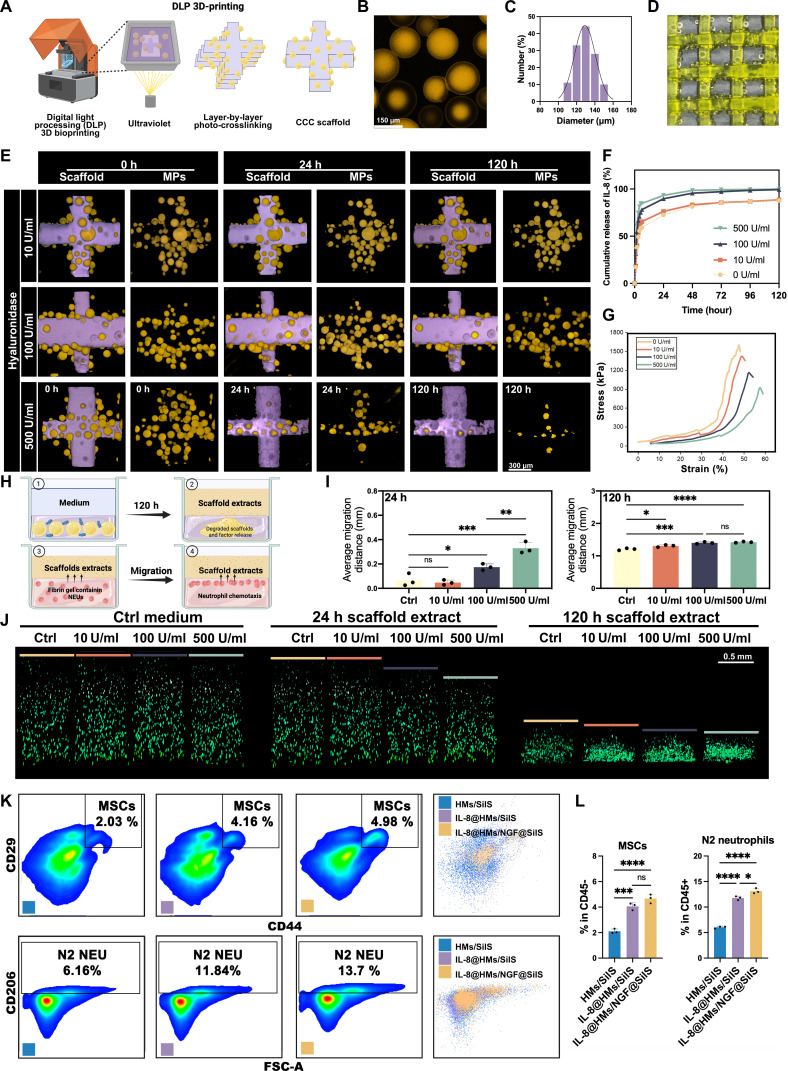
Timely reviving neutrophil chemotaxis and sensory innervation for promoting MSC recruitment. (A) Schematic illustration of CCC scaffold preparation. (B) Monodispersed HAMA MPs and (C) particle size distribution. Scale bars, 150 μm. (D) Image of scaffold observed under a stereomicroscope. (E) 3D confocal reconstructions showing the degradation of HAMA MPs (yellow) with varying hyaluronidase concentrations within the scaffold matrix (purple). Scale bars, 300 μm. (F) Cumulative release of IL-8 from HAMA MPs with varying hyaluronidase concentrations within the scaffold matrix. (G) Compression stress–strain curve of the scaffolds. (H) Schematic diagram of the neutrophil chemotaxis experiment in fibrin gel. (I) Average migration distance and (J) representative images of neutrophil chemotaxis in fibrin gel. Scale bars, 0.5 mm. (K and L) Flow cytometry analysis of N2 neutrophil polarization and MSC recruitment. Data are mean ± SEM analyzed by one-way ANOVA and post hoc multiple comparisons; ns indicates no statistically significant difference; *P < 0.05, **P < 0.01, ***P < 0.001, ****P < 0.0001.

To rescue the disrupted spatiotemporal inflammation pattern and degenerated nerve caused by DM, we fabricated a microsphere (MP)-exposed CCC scaffold with biomimetic spatiotemporal cytokine release using DLP printing. In conventional extrusion-based 3-dimensional (3D) bioprinting, MPs must be substantially smaller than the nozzle diameter to avoid rupture from shear stress [43], and are typically embedded within the scaffold interior [44]. In contrast, the projection-based printing of DLP frees the MP size from the constraints of the nozzle. By tuning layer thickness, MP diameter, and scaffold design, we directly produced an MP-exposed CCC structure from MP-laden bioink (Fig. 2A). MPs were designed with a diameter of ~130 μm (Fig. 2B and C), producing pores of comparable size upon degradation—a dimension known to promote bone formation [45]. To match the MP size, scaffold struts were set to 400 μm in diameter (Fig. 2D). Hyaluronic acid methacrylate (HAMA) microspheres (HMs) were fabricated via a microfluidic aqueous/oil method [46], with droplet size controlled by the diameter of microfluidic channels and the flow rate ratio of oil (QO) to aqueous phase (QA) [46]. The layer-by-layer curing mechanism of DLP printing requires the scaffold matrix to maintain sufficient mechanical strength to prevent collapse. Silk fibroin was selected for its favorable strength, biocompatibility, cell adhesion, and degradation kinetics compatible with tissue regeneration according to our previous studies [47–51]. To enhance printing precision, 5% polyethylene glycol diacrylate (PEGDA) was incorporated into the matrix containing 10% SilMA as scaffold matrix (SilS). HM-contained bio-ink was prepared at a 1:10 mass ratio of HMs to SilS, as previously described [52].

IL-8 is a potent chemokine that drives neutrophil chemotaxis and activation. It also promotes neutrophil N2 polarization, which enhances their capacity to recruit MSCs and initiate downstream regenerative processes [53,54]. NGF serves as a key neuroregenerative and reinnervation signal within bone [55–59]. Our previous work showed that excessive inflammation and impaired ossification in diabetic alveolar bone defects are closely associated with reduced Ngf expression [38]. Therefore, IL-8 was loaded in HAMA MPs (IL-8@HMs) for rapid release and early neutrophil infiltration, while NGF was loaded in SilMA-contained scaffold matrix (NGF@SilS) for sustained release and nerve ingrowth. Recombinant IL-8 (80 μg/ml) was incorporated into the HAMA precursor solution to achieve optimal neutrophil-mediated MSC recruitment (Fig. S2A to C). Recombinant NGF (100 μg/ml) was added to the SilS precursor solution to promote sensory nerve reinnervation (Fig. S2D and E).

To recapitulate the early neutrophilic dynamics in diabetic defects, hyaluronidase was incorporated into the SilS bio-ink to ensure rapid and complete IL-8 release prior to the physiological resolution of inflammation. Specifically, MP degradation in scaffold matrix containing 10, 100, or 500 U/ml hyaluronidase was monitored via 3D confocal imaging. Minimal degradation was observed at 10 U/ml, whereas both 100 and 500 U/ml groups achieved substantial MP degradation by 120 h (Fig. 2E). Supernatants were collected at predefined intervals, and IL-8 concentrations were quantified by enzyme-linked immunosorbent assay (ELISA) to determine release kinetics (Fig. 2F). Cumulative release in the 0 and 10 U/ml groups remained below 90%, whereas the 100 and 500 U/ml groups reached ~99% by 120 h. Notably, the 500 U/ml group exhibited excessive MP degradation, which compromised the scaffold’s compressive strength (Fig. 2G). To further assess how hyaluronidase-regulated IL-8 release influences neutrophil migratory speed, neutrophils were embedded within a fibrin gel, and their migration induced by scaffolds extracts collected at different time points was quantified following the method of Liu and colleagues [60] (Fig. 2H). To simulate post-implantation factor release and align it with the physiological timeline of neutrophil influx during bone healing, scaffold extracts collected at 24 h (the onset of neutrophil chemotaxis in vivo) and 120 h (the peak of neutrophil infiltration) were assessed for their ability to promote neutrophil chemotaxis. As shown in Fig. 2I and J, in the 24-h extracts, both the 100 and 500 U/ml groups induced significant neutrophil migration. In the 120-h extracts, neutrophil migration became more pronounced in these 2 groups, and the migration distances between them showed no significant difference. Considering that rapid degradation in the 500 U/ml group compromised mechanical strength of the scaffolds, we ultimately selected 100 U/ml as the optimal concentration for subsequent studies.

As described above, insufficient early neutrophil infiltration in diabetic defects may compromise MSC recruitment, thereby delaying the initiation of regeneration. N2-polarized neutrophils, which expressed arginase-1 and mannose receptor (CD206), were responsible for pro-reparative bioactivities, such as MSC recruitment and angiogenesis [40,61–64]. Although IL-8 is known to promote N2 neutrophil polarization, our previous work also showed that sensory denervation reduces both N2 neutrophils and stem cell proportion. Therefore, a Transwell assay was employed to determine whether sensory neurons stimulated by the NGF-loaded CCC scaffold could enhance MSC recruitment. As indicated in Fig. S2F and G, MSCs isolated from diabetic mice exhibited limited migratory capacity. Coculture with DRG neurons or the NGF-loaded CCC scaffold, or both, failed to significantly enhance MSC migration, whereas coculture with neutrophils partially rescued MSC recruitment. However, when NGF-stimulated DRG neurons were cocultured with neutrophils, MSC recruitment was markedly enhanced compared to group cocultured with only neutrophil, indicating that the MSC-recruiting effect of sensory neurons may act through neutrophils. Furthermore, the different scaffolds were implanted into femoral defects in diabetic mice (Fig. S2H). The proportion of N2 neutrophils and MSCs was quantified by flow cytometry at 120 h. The IL-8-loaded CCC scaffold significantly increased the proportion of MSCs (CD45^−^CD44^+^CD29^+^). The additional loading of NGF further elicited a slight, though statistically insignificant, elevation in MSC proportions (Fig. 2K and L). Likewise, N2-polarized neutrophils (CD45^+^Ly6G^+^CD206^+^) were also increased following NGF addition (Fig. 2K and L). Taken together, such interaction between sensory neurons and neutrophils constituted a critical step in the onset of bone regeneration.

### NGF released from CCC scaffold enhanced sensory neuron-mediated osteogenesis

**Fig. 3.**
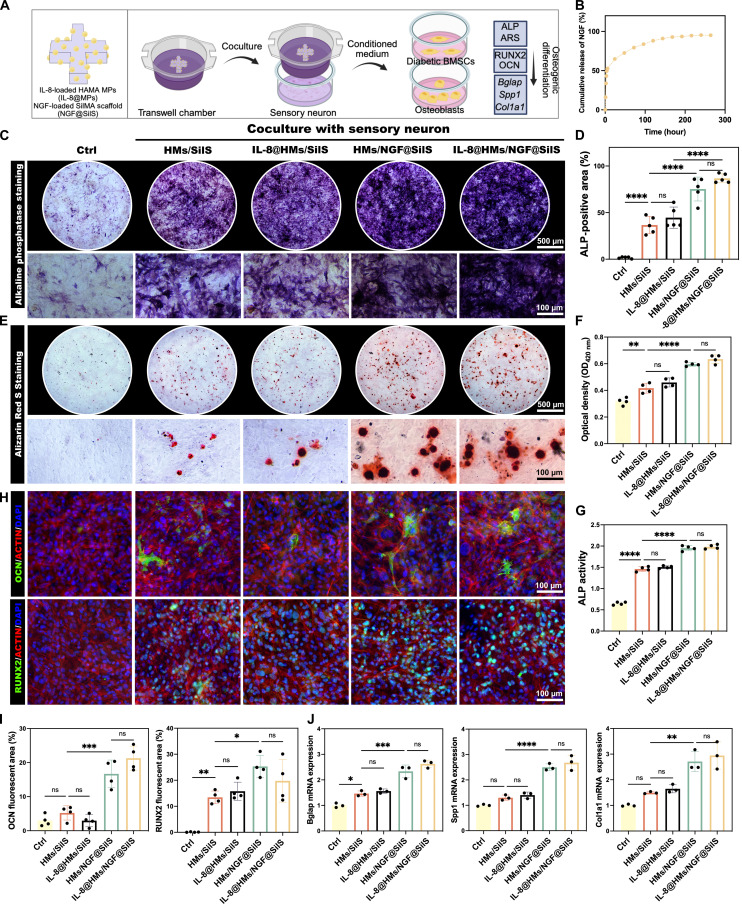
NGF released from CCC scaffolds enhanced sensory nerve-mediated osteogenesis. (A) Experimental design schematic. (B) Cumulative release of NGF from the scaffold matrix. (C and D) Representative images and quantitative analysis of ALP staining of BMSCs with indicated CM for 7 d (scale bars, 500 or 100 μm). (E and F) Representative images and quantitative analysis of Alizarin Red S (ARS) staining for 14 d (scale bars, 500 μm or 100 μm). (G) ALP activity assay of BMSCs (*n* = 4). (H and I) Representative immunofluorescent staining images and quantitative analysis of BMSCs (*n* = 4; scale bars, 100 μm). (J) RT-PCR analysis of osteogenic-related gene expression (Bglap, Spp1, Col1a1) in BMSCs. Data are mean ± SEM analyzed by one-way ANOVA and post hoc multiple comparisons; ns indicates no statistically significant difference; *P < 0.05, **P < 0.01, ***P < 0.001, ****P < 0.0001.

How to direct the osteogenic differentiation of recruited MSCs is pivotal to bone healing. Sensory nerves contribute to bone repair through several mechanisms, including the promotion of osteogenic differentiation [65–71]. To assess the pro-regenerative effect of the CCC scaffolds on sensory-innervated bone healing, an indirect coculture system was established using sensory neurons and bone marrow-derived mesenchymal stromal cells (BMSCs) isolated from diabetic mice (Fig. 3A). Prior to coculture, supernatants from CCC scaffolds were collected at defined intervals, and NGF concentrations were quantified by ELISA (Fig. 3B). NGF release reached approximately 90% by 168 h and sustained for up to 264 h. Alkaline phosphatase (ALP) staining (Fig. 3C and D) and ALP activity assays (Fig. 3G) showed that after 7-d incubation, BMSCs, which were cultured with conditioned medium (CM) from sensory neurons treated with NGF-containing scaffolds (HMs/NGF@SilS or IL-8@HMs/NGF@SilS), exhibited the most obvious osteogenic differentiation in all groups. CM from sensory neuron–HMs/SilS coculture system also induced a moderate but significant increase in ALP activity relative to HMs/SilS scaffolds alone. Alizarin Red staining confirmed greater mineral deposition after 14 d (Fig. 3E and F), with NGF-loaded scaffolds—regardless of IL-8 incorporation—inducing larger and denser mineralized nodules. Immunofluorescence of RUNX2 and OCN (Fig. 3H and I), together with quantitative polymerase chain reaction (qPCR) analysis of late osteogenic markers (Bglap, Spp1, Col1a1), further verified that NGF-loaded CCC scaffolds augmented sensory nerve-mediated osteogenesis (Fig. 3J).

### Neuroimmune synapse-like structure restores macrophage efferocytosis

**Fig. 4.**
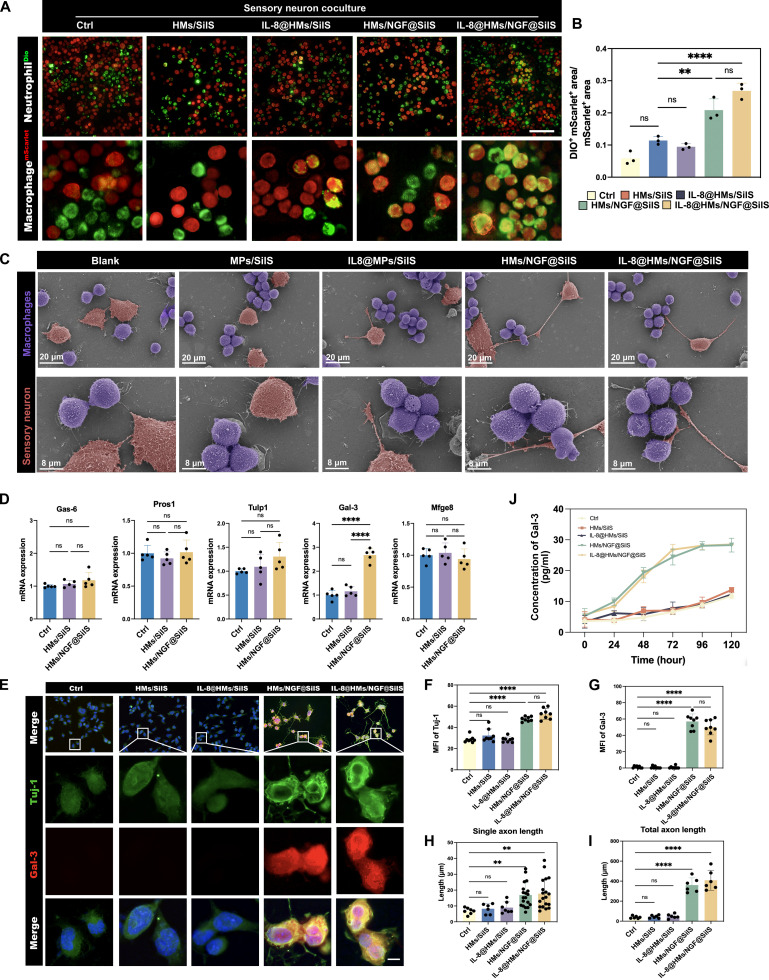
Neuroimmune synapse-like structure restores macrophage efferocytosis. (A) Representative images and (B) semiquantitative assessment of efferocytosis observed in coculture of mScarlet-labeled macrophages and DIO-labeled neutrophils, following 72-h incubation in indicated groups. Scale bar, 100 μm. (C) Representative SEM images of macrophages and sensory neurons cocultured in indicated groups. Scale bars, 20 and 8 μm. (D) Gene expression of efferocytic ligands was assessed in indicated groups (*n* = 5). (E to G) Representative images and quantitative analysis of DRG neuron cocultured with the indicated scaffolds for 120 h, immunofluorescently stained for Tuj-1 and Gal-3 (*n* = 3; scale bars, 10 μm). (H and I) Quantitative analysis of neurite outgrowth. (J) Gal-3 levels in the supernatant of DRG neurons cocultured with the indicated scaffolds for 120 h (*n* = 3). Data are mean ± SEM analyzed by one-way ANOVA and post hoc multiple comparisons; ns indicates no statistically significant difference; *P < 0.05, **P < 0.01, ***P < 0.001, ****P < 0.0001.

Clearance of apoptotic neutrophils by macrophage efferocytosis, which was impaired under hyperglycemia condition, was necessary for inflammation resolution. Considering that neuroimmune interplay between sensory neuron and neutrophils promoted MSC migration, we further tested whether promoting sensory innervation with CCC scaffolds could restore macrophage efferocytosis. Firstly, neutrophils and macrophages were isolated from the bone tissue of diabetic and control mice. Then, neutrophils were labeled with DIO, and macrophages were transfected with mScarlet. The 2 cells were cocultured with DRG neurons to assess macrophage efferocytosis. Fluorescence imaging showed that increased red–green colocalization only took place in NGF-loaded and IL-8/NGF-loaded CCC scaffolds, indicating that NGF-activated sensory neurons substantially improved efferocytosis (Fig. 4A and B).

Scanning electron microscopy (SEM) was further performed to dissect the macrophage–sensory neuron direct interaction under different scaffold conditions. In the control and HMs/SilS groups, neurons and macrophages were evenly distributed, with no discernible neurite extension or cell–cell contact. IL-8-loaded scaffolds induced macrophage aggregation, but the cells remained spatially separated from neurons. In contrast, the NGF-loaded scaffold induced significant neurite outgrowth, and macrophages preferentially localized to neuronal somas and outgrowing neurites, forming membrane–membrane contacts (Fig. 4C). Transmission electron microscopy (TEM) was employed to examine the contact sites between sensory neuron and macrophage, where typical synaptic vesicles and dense-core vesicles previously reported [33] were observed in pre-synapse area (Fig. S3A). These results suggested that the neuroregulatory effect on macrophage efferocytosis might be mediated through this neuron–macrophage synapse-like interface.

Mechanistically, efferocytosis is also driven by the recognition of “eat-me” signals on apoptotic cell membrane by receptors on phagocytes, which motivated us to identify potential ligand–receptor interaction between neurons and macrophages. In addition to the direct recognition of phosphatidylserine (PS) by phagocytes, various secreted proteins act as bridging molecules that bind PS and activate receptor-driven efferocytosis. Representative genes encoding efferocytosis ligands include Gas6, Pros1, Tulp1, and Gal-3, which bind TAM receptors [72], as well as Mfge8, which cooperates with integrin receptors (e.g., αvβ3/β5) [73]. We assessed all 5 ligands in diabetic DRG neurons with or without NGF stimulation by reverse transcription PCR (RT-PCR). Only the Gal-3 showed a significant up-regulation in response to NGF, increasing 2.67 ± 0.28-fold compared with the control (Fig. 4D). DRG neurons were further cocultured with the CCC scaffold to evaluate their neurogenic responses, including neurite outgrowth and Gal-3 production (Fig. S4A). Immunofluorescence staining showed markedly increased expression of both Tuj-1, a neuronal microtubule cytoskeletal marker, and Gal-3 in the NGF-loaded groups, whereas neither of the expression of Tuj-1 nor Gal-3 was influenced by IL-8 (Fig. 4E to G). Quantitative analysis of neurite extension demonstrated that NGF-loaded groups significantly increased both the average axon length per neuron and the total neurite length (Fig. 4H and I). Colocalization analysis revealed that Gal-3 was synthesized in Tuj-1^high^ DRG neurons and localized in both the cytoplasm and nucleus (Fig. S4B). More important, the expression level of Gal-3 in the NGF-loaded groups was elevated to nearly 3-fold of that in the blank control (Fig. S4C). Supernatants from DRG–scaffold cocultures were sequentially collected over 120 h and analyzed for Gal-3 concentration (Fig. 4J). NGF-induced Gal-3 secretion gradually increased from 0 to 72 h, peaking at approximately 30 pg/ml between 72 and 120 h. Interestingly, a mild elevation of Gal-3 was also observed in control or NGF-free scaffold groups, which may reflect basal expression in resting DRG neurons. Gal-3 functions as a ligand for the MerTK receptor, facilitating efferocytosis by bridging apoptotic cells to phagocytic receptors on macrophages [74,75] and by activating the classical “eat-me” signal on neutrophils [76]. The administration of MerTK inhibitor UNC2541 inhibited the pro-efferocytosis ability of CCC scaffold-activated DRG neurons (Fig. S4D). Then, the Gal-3 gene in DRG neurons was also knocked down through small interfering RNA (siRNA) in vitro (Fig. S4E). Silencing Gal-3 significantly attenuated macrophage efferocytosis induced by sensory neurons (Fig. S4F and G). These results underscore the importance of Gal-3/MerTK axis in such neuroimmune interplay. Collectively, these findings demonstrate that the NGF-loaded CCC scaffold effectively activated sensory neurons and enhanced their production of Gal-3, which promoted macrophage-mediated clearance of apoptotic neutrophils to accelerate the inflammation resolution.

In addition, efferocytosis has been reported to promote macrophage M2 polarization and tissue regeneration [77]. Therefore, the effects of neuron-induced efferocytosis on macrophage polarization have also been evaluated under the same condition. As indicated in Fig. S5A and B, CM harvested from DRG cocultured with IL-8/NGF-loaded CCC scaffolds modestly increased M2 polarization, whereas apoptotic cells without scaffolds also enhanced this effect. Notably, the combination of factor-stimulated neuronal CM and apoptotic cells induced the strongest M2 polarization. Interestingly, the IL-8/NGF-loaded scaffold group increased the proportion of M1 macrophages, but this effect disappeared in the presence of apoptotic cells, indicating that efferocytosis can override pro-inflammatory polarization. In addition, the apoptotic cells + IL-8/NGF CCC scaffold further enhanced the expression of osteogenesis-related genes while promoting M2 polarization in macrophages cocultured with sensory neuron. (Fig. S5C).

To further investigate the mechanism of IL-8/NGF-loaded CCC scaffold-mediated efferocytosis in macrophages, we conducted bulk mRNA sequencing to compare the transcription profile in macrophages after 24-h stimulation of CM, referred to as Treat, with the control group (Ctr) (Fig. S6A). The RNA-sequencing data showed well-separated gene expression patterns between 2 groups with a total of 673 down-regulated and 760 up-regulated genes (*P* < 0.05) (Fig. S6B to D). The up-regulated GO biological process terms are intensely enriched on regeneration- and efferocytosis-related processes, especially phagocytosis (Fig. S6E). Gene set enrichment analysis (GSEA) further revealed that the phagosome-related genes were significantly enhanced under the stimulation of CM (Fig. S6F). The protein–protein interaction (PPI) analysis further identified *Actb* as the crucial hub gene among all phagocytosis-related genes (Fig. S6G and H). β-Actin (encoded by *Actb*) plays a fundamental structural role in efferocytosis by polymerizing into filamentous actin that drive phagocytic cup formation and apoptotic cell engulfment [78]. Notably, siRNA-mediated suppression of neuronal Gal-3 synthesis, as well as pharmacological inhibition of MerTK using UNC2541, both attenuated *Actb* expression, indicating that the transcriptional regulation of *Actb* is governed by the Gal-3/MerTK axis (Fig. S6I).

### CCC scaffold reprograms diabetic inflammatory microenvironment in mandibular bone defects

**Fig. 5.**
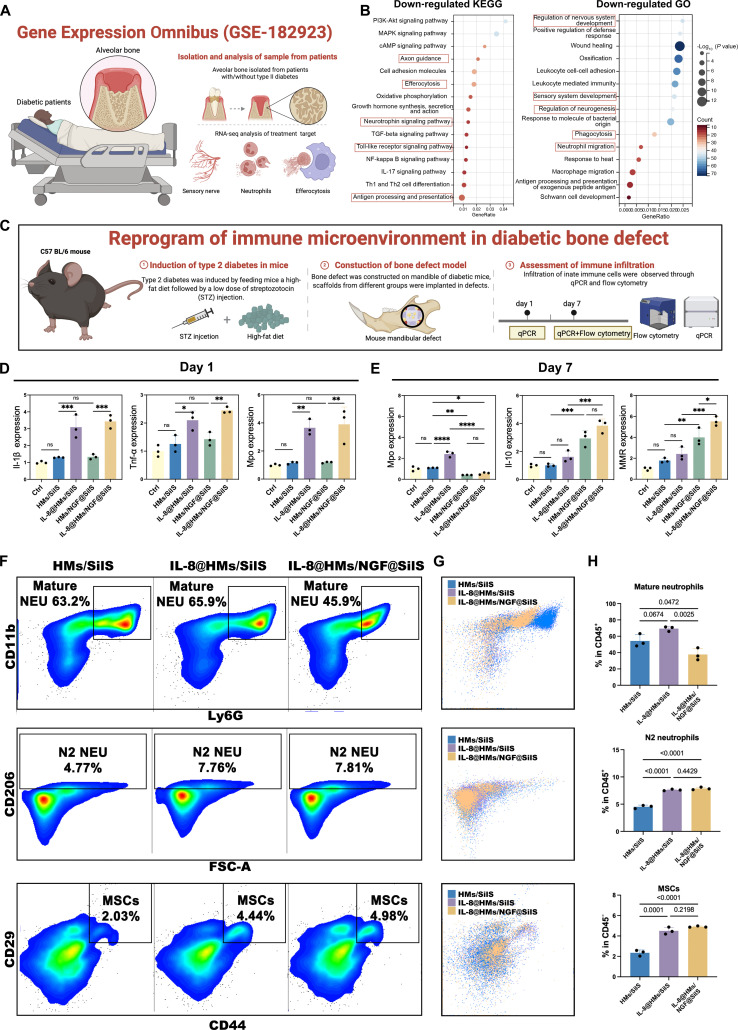
CCC scaffold reprograms diabetic inflammatory microenvironment in mandibular bone defects. (A) Schematic representation of transcriptome data analysis of RNA-seq in GEO database. (B) GO and KEGG enrichment analysis of DEGs. (C) Experimental design schematic. (D) RT-PCR analysis of inflammation-related gene expression (Il-1β, Tnf-α, and Mpo) and (E) resolution-related gene expression (Il-10 and MMR). (F to H) Flow cytometry analysis of neutrophilic inflammation and MSC recruitment. Data are mean ± SEM analyzed by one-way ANOVA and post hoc multiple comparisons; *P < 0.05, **P < 0.01, ***P < 0.001, ****P < 0.0001. Other *P* values were annotated in the figure.

To validate that such neuroimmune interaction is a universal immunoregulatory mechanism of different bone defects, we explored the neuroimmune interplay in diabetic oral–maxillofacial bone defects. Firstly, we reanalyzed the transcriptional profiles of alveolar bone from diabetic and healthy individuals (GSE182923, Ayilavarapu et al.[79]) (Fig. 5A and B). GO and KEGG enrichment analyses revealed significant impairments in neutrophil migration. Concurrently, phagocytic pathways required for inflammation resolution, including efferocytosis-related biological processes, were markedly down-regulated (Fig. 5B). Notably, among the down-regulated differentially expressed genes (DEGs), sensory innervation-related biological processes were also significantly suppressed in diabetic alveolar bone. Together, these results indicate that the neuro–immune axis is likewise disrupted in the mandibular bone under diabetic conditions.

To assess whether the CCC scaffold could sequentially reprogram the inflammatory microenvironment during oral–maxillofacial bone regeneration, a mandibular defect model was established in diabetic mice (Fig. 5C). Scaffolds from each experimental group were implanted into the defects, and mandibular tissues were collected at 1 d (acute inflammatory phase) and 7 d (early regenerative phase) post-surgery for RT-PCR analysis. *Il-1β* and *Tnf-α*, classical markers of acute inflammation, were examined to evaluate early immune activation. As shown in Fig. 5D, the blank scaffold elicited only a slight, nonsignificant increase in these cytokines. The IL-8-incorporated scaffolds (IL-8@HMs/SilS and IL-8@HMs/NGF@SilS) displayed marked elevation of *Il-1β* and *Tnf-α*, as well as up-regulation of neutrophilic marker myeloperoxidase (*Mpo*), indicating effective early neutrophilic infiltration. Contrarily, minimal inflammatory responses were observed in the HMs/NGF@SilS group within 24 h. At 168 h (day 7) post-implantation, *Mpo* and *Il-10* expression was examined to assess ongoing neutrophil-related inflammation and anti-inflammatory signaling (Fig. 5E). The blank scaffold (HMs/SilS) had minor influence on the inflammatory profile of the diabetic microenvironment. The IL-8@HMs/SilS group showed sustained elevation of *Mpo*, whereas no significant difference in *Il-10* levels between this group and blank group was observed, suggesting impaired inflammation resolution and IL-8-induced persistent immune activation. Notably, the NGF-loaded scaffolds (HMs/NGF@SilS) reduced *Mpo* expression by approximately 50% at day 7—a suppressive effect not observed at 24 h—implying a delayed anti-inflammatory response potentially mediated by sensory innervation. Moreover, *Il-10* expression in this group increased by 3- to 4-fold relative to the blank scaffold, further supporting enhanced anti-inflammatory signaling. In parallel, NGF delivery significantly up-regulated the expression of macrophage mannose receptor (MMR), a marker of M2 macrophage polarization (Fig. 5E).

Flow cytometric analysis confirmed the trends observed in gene expression data (Fig. 5F to H). Mature neutrophils (CD45^+^ Ly6G^+^ CD11b^+^), which are characterized by elevated reactive oxygen species (ROS) production and enhanced NET formation, possess stronger pro-inflammatory potential compared to immature subsets [80,81]. In contrast, N2 neutrophils (CD45^+^CD11b^+^CD206^+^) represent a reparative subpopulation associated with inflammation resolution and tissue regeneration [62,63,82–84]. Accordingly, we quantified the proportions of mature and N2 neutrophils 7 d after scaffold implantation. Similar to aforementioned results, the IL-8@HMs/SilS scaffold induced a moderate, though statistically insignificant, increase in the proportion of mature neutrophils (CD45^+^ Ly6G^+^ CD11b^+^) relative to the control group, due, at least in part, to the chemotactic ability of IL-8. In line with previous reports [40], IL-8 delivery also promoted an increase in the proportion of N2 neutrophils (CD45^+^CD11b^+^CD206^+^). Notably, co-delivery of NGF (IL-8@HMs/NGF@SilS) effectively attenuated the accumulation of mature neutrophils while further enhancing the N2 neutrophil population, indicating improved immunomodulatory efficacy. In addition, MSCs (CD45^−^CD29^+^CD44^+^) were quantified to evaluate the scaffold’s pro-regenerative potential. IL-8/NGF co-delivery significantly increased MSC infiltration. Collectively, the biomimetic spatiotemporal delivery of IL-8 and NGF via the CCC scaffold achieves a biomimetic reprogramming of the inflammatory microenvironment in diabetic bone defects, restoring the temporal balance between pro-inflammatory activation and inflammation resolution, thereby enhancing regenerative outcomes.

### CCC scaffold restored neutrophil fate and neuroimmune interface to enhance bone healing in rats

**Fig. 6.**
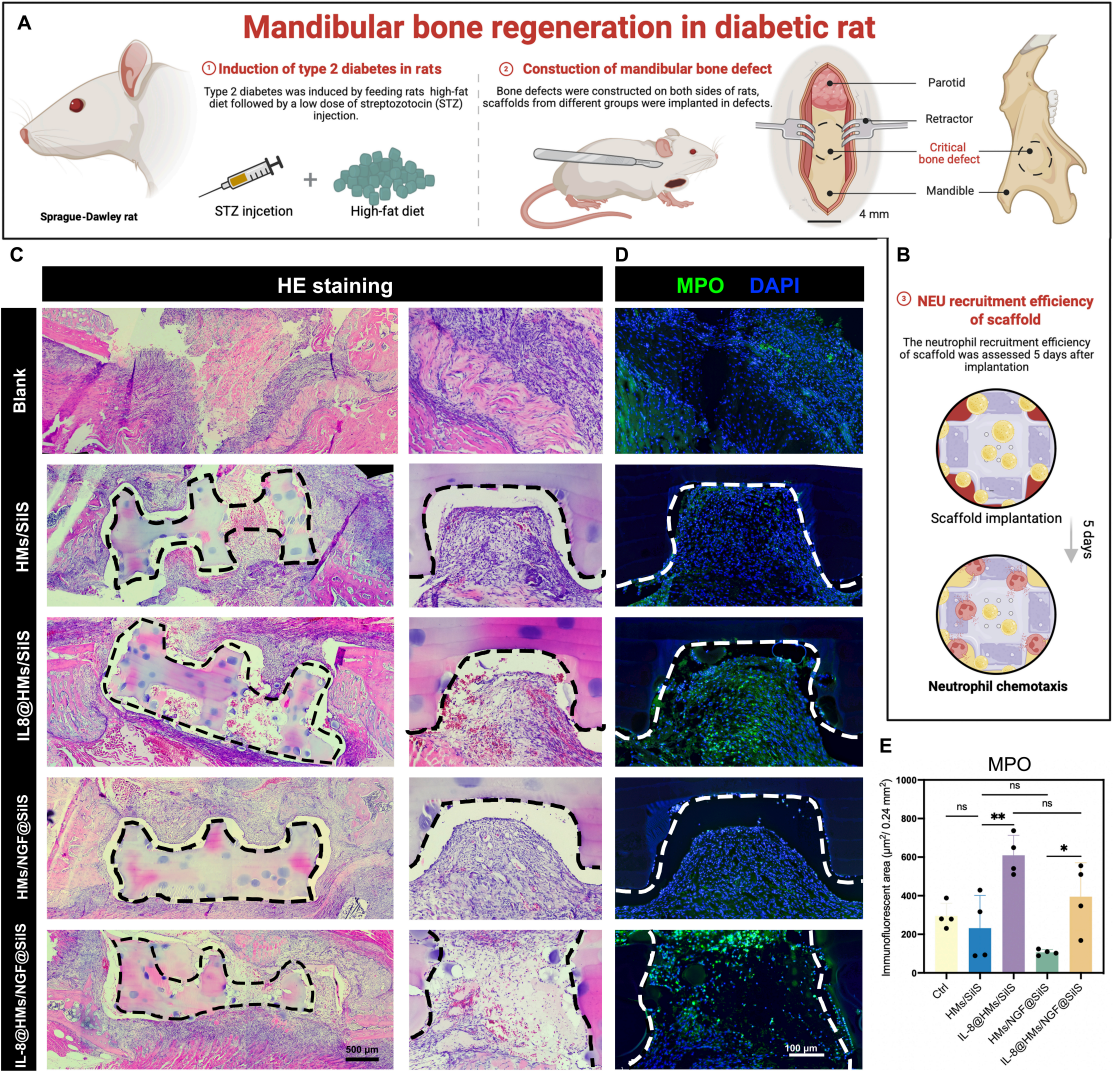
Histological assessment of CCC scaffold-induced neutrophilic inflammation at early stage of diabetic bone healing. (A and B) Experimental design schematic. (C and D) Representative images of H&E staining (left), high-magnification images (middle), and immunostaining (right) of the neutrophil immunohistochemically stained for myeloperoxidase (MPO) (green)/DAPI (blue) 5 d after scaffold implantation. The dashed lines indicate the boundary of scaffolds. Scale bars, 500 or 100 μm. (E) Semiquantitative analysis of MPO-positive area. Data are mean ± SEM analyzed by one-way ANOVA and post hoc multiple comparisons; ns indicates no statistically significant difference; *P < 0.05, **P < 0.01, ***P < 0.001, ****P < 0.0001.

To assess the generalizability across species and defect sites, as well as the efficacy of CCC scaffolds in repairing larger critical-sized mandibular defects, we further transitioned to a diabetic rat model to comprehensively evaluate the long-term therapeutic efficacy and osteogenic potential of the CCC scaffold in repairing critical-sized mandibular defects, and an in vivo study was performed to assess its immunomodulatory effects and osteogenic potential. Standardized surgical procedures were employed to create 4-mm-diameter defects in the rat mandible [85]. Rats with untreated defects served as the blank group, while the remaining animals were assigned to experimental groups based on the type of implanted scaffold (Fig. 6A and B).

**Fig. 7.**
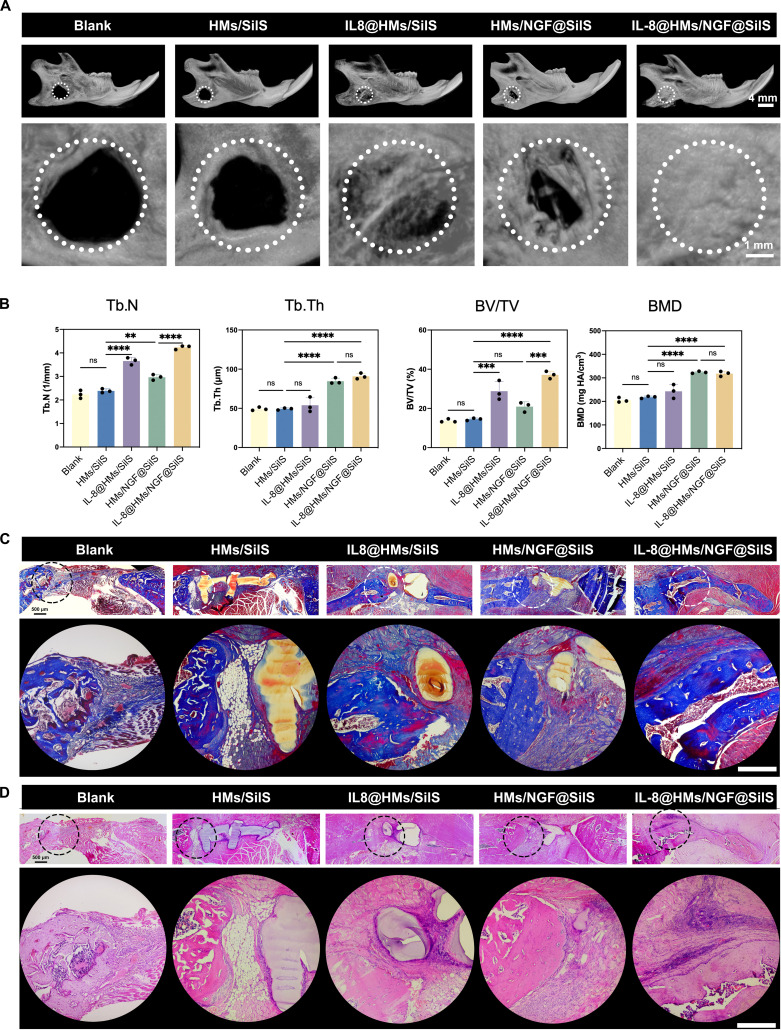
Histological assessment of bone regeneration induced by the CCC scaffold in diabetic mandibular defects. (A) Micro-CT reconstructions and (B) quantitative analysis of bone volume/total volume (BV/TV), trabecular number (Tb.N), trabecular thickness (Tb.Th), and bone mineral density of rat mandible on 8 weeks post-injury(*n* = 5 mandibles per group). Scale bars, 4 and 1 mm. (C) Representative images of Masson’s trichrome staining and (D) H&E staining. Scale bars, 500 μm. Data are mean ± SEM analyzed by one-way ANOVA and post hoc multiple comparisons; ns indicates no statistically significant difference; *P < 0.05, **P < 0.01, ***P < 0.001, ****P < 0.0001.

Five days after implantation, rat mandibles were collected for H&E and immunofluorescent staining to evaluate neutrophilic inflammation (Fig. 6C to E). In the blank group, only soft tissue was observed in the defect area. Limited neutrophil infiltration was observed in the blank and the factor-free group, consistent with previous findings in mouse femoral defects, indicating a delayed neutrophil response. IL-8-loading scaffolds induced obvious neutrophilic influx at the injured sites despite the presence of NGF, which might result from the delayed onset of sensory ingrowth. Samples were collected for radiological and histological analysis to evaluate bone formation 8 weeks after implantation. As shown in Fig. 7A and B, compared to the blank and HMs/SilS groups, the IL-8@HMs/SilS and HMs/NGF@SilS scaffolds increased the new bone formation to some extent: The former exhibited insufficient trabecular thickness in the newly formed bone, while the latter showed delayed bone formation at the center of the defects. As anticipated, the dual-factor-loaded “CCC” scaffold (IL-8@HMs/NGF@SilS) overcame the limitations of both single-factor scaffolds, achieving complete repair of the critical-sized bone defect within 8 weeks. Histological analysis also revealed similar results (Fig. 7C and D). In the HMs/SilS group, a large amount of undegraded scaffold residues was observed, surrounded by inflammatory cell infiltration. Increased osteoid-like tissue, exhibiting light blue in Masson stain, was observed in IL-8@HMs/SilS and HMs/NGF@SilS groups, which indicated enhanced bone formation. Notably, in the IL-8@HMs/NGF@SilS group, the newly formed bone completely bridged the defect.

**Fig. 8.**
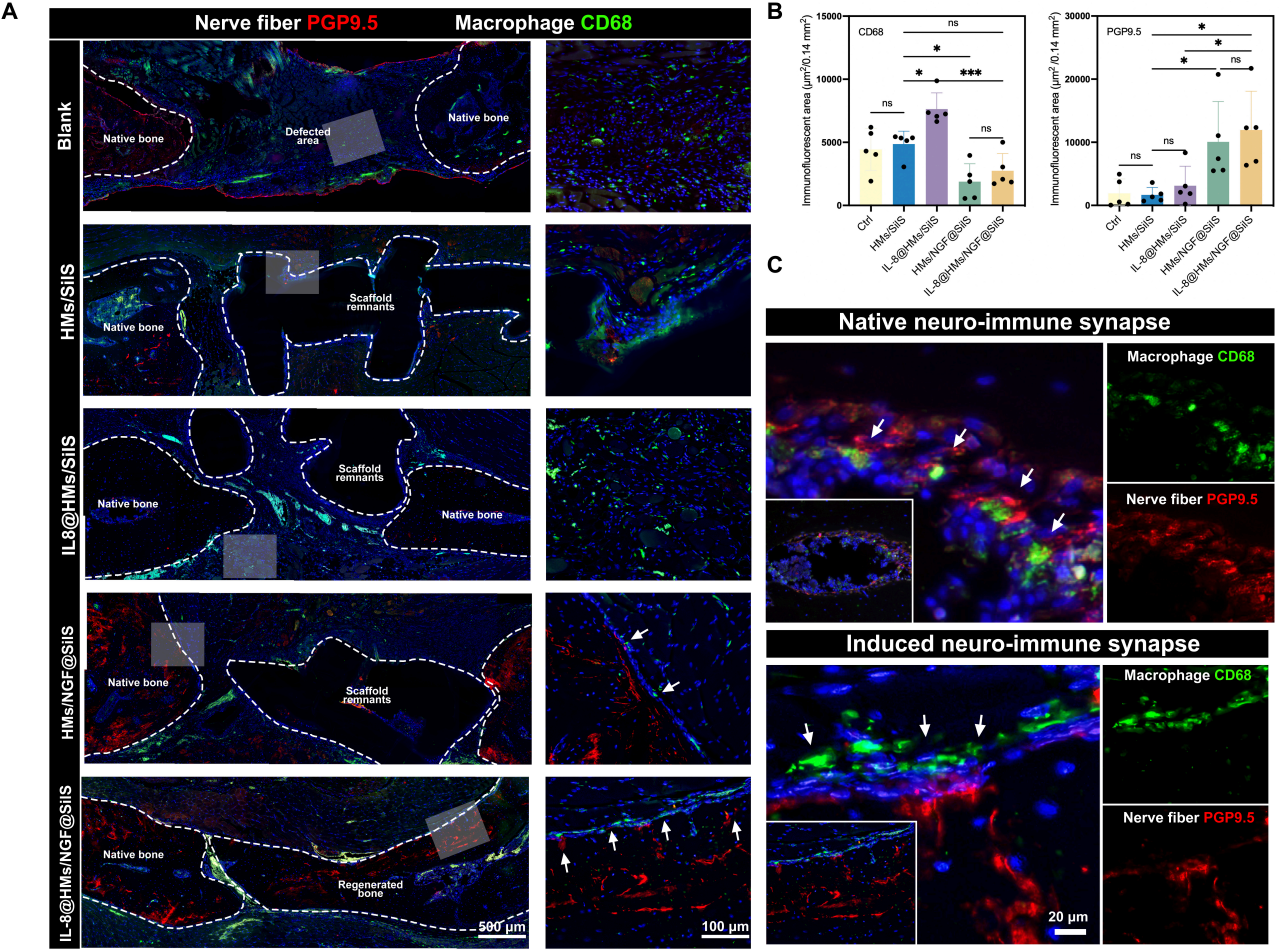
NGF-loaded CCC scaffolds induce synapse-like structures between macrophages and sensory nerves. (A) Representative images and (B) quantitative analysis of CD68^+^ macrophages and PGP9.5^+^ nerve fibers. Scale bars, 500 and 100 μm. (C) Representative images of neuro–immune synapse observed in native bone or regenerated bone induced by IL-8@HMs/NGF@SilS. Scale bars, 20 μm. Data are mean ± SEM analyzed by one-way ANOVA and post hoc multiple comparisons; ns indicates no statistically significant difference; *P < 0.05, **P < 0.01, ***P < 0.001, ****P < 0.0001.

Immunofluorescence staining was further performed to evaluate the neuro–immune interface between macrophage (labeled by CD68) and sensory nerve fiber (labeled by PGP9.5 and CGRP) in the 8-week samples (Fig. 8A and B and Fig. S7A and B). The most pronounced CD68-positive signal was observed in the tissues adjacent to scaffold remnants in the IL-8@HMs/SilS group, further confirming the stimulation of IL-8 on innate immune responses, while the blank and HMs/SilS groups exhibited a more diffuse distribution of macrophages throughout the defect area, consistent with the chronic, unresolved inflammation characteristic of diabetic bone healing. Notably, only a sparse distribution of macrophages was observed at the osteogenic interface in NGF-loaded groups (HMs/NGF@SilS and IL-8@HMs/NGF@SilS), indicating successful immunomodulation by nerve innervation in the defect area. For nerve ingrowth, immunostaining revealed sparse nerve fiber distribution limited to the wound margin or adjacent native bone in the blank and NGF-free scaffold groups, indicating insufficient reinnervation under diabetic conditions. In contrast, NGF delivery not only substantially increased nerve fiber density within the native bone at the defect boundary but also resulted in extensive nerve ingrowth in the newly formed bone tissues. Moreover, in uninjured native bone, a small subset of macrophages was closely apposed to PGP9.5^+^ nerve fibers, indicating the presence of an endogenous synapse-like neuroimmune interface (Fig. 8C). Such structures were scarcely detectable in regenerated tissue, except for the NGF-loaded groups. Specifically, at the edge of the newly formed bone in NGF-loaded CCC scaffolds, these synapse-like interactions were re-established (Fig. 8C). Although the density of such contacts was lower than that in native bone, the similar spatial organization and comparable macrophage numbers suggested the restoration of functionally analogous neuro–immune interfaces.

In addition, nerve ingrowth and vascular formation are spatially and functionally coordinated during bone regeneration, a process referred to as neuro–vascular coupling, which is essential for effective bone repair [86]. We therefore evaluated angiogenesis in defected mandibles in diabetic rats. In the blank group, minimal vascular formation was observed surrounding the nonregenerated bone interfaces, and implantation of factor-free scaffolds failed to improve this condition. The IL-8@HMs/SilS scaffold resulted in a modest increase in CD31^+^ vascular areas. In contrast, NGF-loaded scaffolds significantly enhanced angiogenesis within the defect region during diabetic bone healing, as evidenced by increased CD31^+^ vessel density and *Vegf* expression (Fig. S8A to C). Mechanistically, the pro-angiogenic capacity of NGF-loaded scaffolds is likely mediated through NGF/TrkA-dependent activation of *Vegf* transcription in sensory nerves [67]. In addition, M2 polarization macrophages induced by efferocytosis have been reported to possess pro-angiogenic properties. Collectively, these direct and indirect effects support the therapeutic potential of neuro-targeted strategies in bone disease treatment.

## Discussion

In this study, we integrated analyses of transcriptomic profiles from diabetic mice and patients with animal models to delineate the altered neutrophil fate throughout diabetic bone healing, which was characterized by delayed but persistent neutrophil infiltration, and impaired macrophage efferocytosis. Notably, inadequate early neutrophil infiltration also impaired stem cell migration, leading to delayed bone healing. Furthermore, reduced sensory innervation was discovered to be associated with this dysregulated neutrophilic inflammation. To harness this deranged immune response, we developed a DLP-printed biomimetic scaffold, which possessed biomimetic spatiotemporal immunomodulation capacity by recovering the neuroimmune crosstalk. Subsequent in vitro and in vivo results highlighted the importance of neuroimmune interactions in orchestrating bone healing process: On the one hand, sensory innervation promoted N2 polarization of neutrophils, facilitating stem cell recruitment to initiate bone regeneration. On the other hand, sensory nerves secreted Gal-3 to activate MerTK receptor on macrophages via a synapse-like structures, thereby restoring the efferocytosis in macrophages to resolve prolonged neutrophilic inflammation.

Inflammation is indispensable for tissue regeneration, yet dysregulated inflammation markedly impairs healing [87]. In diabetes, excessive neutrophil infiltration and aberrant formation of NETs have been widely recognized as major causes of chronic, nonhealing wounds [88,89]. However, the pro-regenerative fate transition of neutrophils has long been underappreciated. Accumulating evidence has recently shed light on the central role of neutrophils in initiating tissue regeneration. Neutrophils are demonstrated to promote ectopic bone formation [40], facilitate muscle repair [14], and enhance alveolar bone regeneration [90]. In this study, we found a pronounced deficiency in early neutrophil infiltration at diabetic bone defects. This early deficit in acute immune response markedly impaired stem cell recruitment and delayed the initiation of bone repair. Importantly, restoring timely neutrophil infiltration via IL-8 reversed these impairments, underscoring the indispensable role of acute neutrophil responses in tissue regeneration. Notably, we further discovered that sensory innervation promoted N2 polarization of neutrophils, thereby enhancing their pro-regenerative capacity and facilitating stem cell recruitment. To our knowledge, such neuroimmune crosstalk in bone regeneration has rarely been described.

The engulfment of apoptotic neutrophils by pro-inflammatory M1 macrophages, followed by their transition toward an M2 reparative phenotype, represents a fundamental step in the inflammation-to-regeneration transition during tissue repair [91]. Nevertheless, under hyperglycemia, down-regulation of crucial receptors for efferocytosis, such as CD36 and class B scavenger type I receptors, significantly hinders the phagocytotic activities of M1 macrophages, resulting in unresolving neutrophilic inflammation [92,93]. By regulating efferocytosis, neutrophil fate can be precisely directed toward inflammation resolution, presenting a promising therapeutic avenue for diabetic wound healing. Yet, achieving cell-specific, long-lasting regulation of efferocytosis still remains challenging [94]. PN is one of the most prevalent diabetic complications, whereas its impact on macrophage efferocytosis has been largely overlooked. Emerging evidence demonstrates that nerves can actively dictate macrophage function to promote tissue repair, suggesting that peripheral nerves may act as promising regulators of immune homeostasis [95–97]. Herein, we identify a previously unrecognized neuroimmune circuit governing macrophage efferocytosis during diabetic bone repair. In vitro and in vivo experiments proved that sensory nerves physically established a synapse-like contacts with macrophages. At this neuroimmune interface, interaction between neuronal Gal-3 and MerTK in macrophages re-activated the phagocytosis of neutrophils. Similar neuroimmune synapse-like structures have been described in the spleen and in tumor microenvironments [30–33], yet none have previously been implicated in bone microenvironment or in the control of efferocytosis. Our results provide mechanistic evidence that this neuroimmune synapse-like interface might function as endogenous mechanism for timely inflammation resolution. Moreover, previous studies revealed that MerTK could stimulate the nucleation of filamentous actin at phagocytic site via the Rac1–Arp axis, thereafter driving engulfment of apoptotic cell [78,98]. In this study, we found that MerTK could also directly up-regulate the expression of *Actb* to enhance efferocytosis.

There are some limitations in our study. Only male mice were used in the present study, leaving the potential effects of sex on the neuroimmune circuit unexplored. The differences in fracture risk between men and women with diabetes [99], as well as the discrepancy in pain perception between sexes [100], indicate the biological relevance of sex differences on neuroimmune interactions in bone. Furthermore, the sympathetic nervous system also plays a key role in bone remodeling [101]. The impact of sympathetic dysfunction on osteo-immunomodulation in diabetes warrants further in-depth investigation.

Collectively, inspired by the feature of the disrupted neutrophilic influx in diabetic bone defects, we designed a CCC scaffold with sequential IL-8 and NGF release to re-establish the coordinated neuroimmune circuit for improved diabetic bone healing. Our findings highlighted a previously unrecognized mechanism by which sensory nerves coordinated early inflammatory resolution and regenerative activation, offering new therapeutic opportunities for diabetes-associated healing disorders.

## Materials and Methods

### Study design

This study aimed to define the spatiotemporal inflammatory landscape of diabetic bone defects and to develop a biomaterial-based strategy to restore neuroimmune coordination for bone regeneration. Based on transcriptomic analyses of bone tissues from diabetic patients and mice, together with in vivo observations in streptozotocin (STZ)-induced diabetic models, we first delineated a characteristic inflammatory pattern marked by lagged yet persistent neutrophilic infiltration and impaired macrophage efferocytosis. Guided by these pathological checkpoints, we engineered a sequential-release “chocolate chip cookie–like” (CCC) scaffold that delivers IL-8 rapidly from surface-exposed MPs and NGF sustainably from a silk fibroin matrix. Mechanistic studies were performed to evaluate IL-8-driven neutrophil recruitment and polarization, neutrophil-mediated MSC chemotaxis, and NGF-dependent sensory nerve regeneration and its regulation of macrophage efferocytosis, with particular emphasis on synapse-like interfaces formed between regenerated axons and macrophages and the involvement of the Gal-3/MerTK pathway. Finally, the therapeutic effects of the CCC scaffold were assessed in full-thickness mandibular defects in diabetic rats randomly assigned to prespecified treatment groups, with nerve ingrowth, immune dynamics, and bone regeneration evaluated at defined time points using immunofluorescence, flow cytometry, micro-computed tomography (CT), and histological analysis.

### Materials

Unless specified, all chemical reagents were purchased from Macklin Co. (Shanghai, China). Fetal bovine serum (FBS), phosphate-buffered saline (PBS), and penicillin/streptomycin were obtained from Gibco-Invitrogen Co. (Grand Island, NY). All consumables were purchased from Corning Inc. (Corning, NY).

### Animals

All mice and rats used in this study were purchased from Shanghai Jiesijie Laboratory Animals Company (China). The care and use of the animals were in strict compliance with the guidelines approved by the Animal Welfare Committee of the Shanghai Jiao Tong University School of Medicine, Affiliated Ninth People’s Hospital (approval number SH9H-2024-A1009-1) and strictly followed the National Institutes of Health Guidelines for the Care and Use of Laboratory Animals.

Type II diabetic models were generated using 8-week-old male C57BL/6 mice or Sprague–Dawley rats according to the established protocol. Initially, the animals were fed a HFD (60 kcal%, FB-12492, WuxiFanbo Biotechnology Co. Ltd., China) for a duration of 4 weeks. Following this dietary intervention, the animals were subjected to an overnight fast and then administered a low-dose injection of STZ (30 mg/kg, S0130, Sigma-Aldrich, Missouri, USA). The successful induction of the type II diabetic state was verified by monitoring fasting blood glucose levels, with a successful model defined as levels consistently exceeding 11.1 mM for 2 consecutive weeks. Upon confirmation of the diabetic model, all animals were transitioned back to a regular diet for subsequent experimental procedures.

### Fabrication and characterization of CCC scaffold

MPs were fabricated using a water-in-oil strategy via a monodisperse MP fabrication system (EFL, China). Briefly, a 3% (w/v) HAMA (EFL, China) solution containing recombinant human IL-8 (Novoprotein, China) was dispersed in corn oil (Sigma, USA) mixed with 1 wt % Span 80 (Sigma, USA). The flow rate of the oil phase (Qo) was 100 μl min^−1^, and the flow rate of the aqueous phase (Qa) was 14 μl min^−1^. The droplets were exposed to ultraviolet radiation at the outlet of the microfluidic device for gelation and then washed with distilled water.

To fabricate the MP-exposed CCC scaffold, a DLP-based 3D printer (EFL, China) was employed to pattern a bio-ink containing suspended MPs. The matrix hydrogel solution consisted of 10% (w/v) SilMA and 5% (w/v) PEGDA. Hyaluronidase (EFL, China) was incorporated to modulate MP degradation speed, and human recombinant NGF (Novoprotein, China) was incorporated to induce sensory nerve reinnervation. The value 1:10 was chosen as the mass ratio of MP/matrix to prepare bio-ink. The light intensity was set to 10 mW/cm^2^, with an exposure time of 10 s per layer. The peeling distance was 6 mm, with a peeling speed of 25 mm/min and a recovery speed of 180 mm/min. The lifting speed was 100 mm/min, and the layer thickness was set to 100 μm. CCC scaffold was observed using a brightfield microscope (Olympus, Japan). To characterize the distribution of MPs in the hydrogel matrix, HAMA and SilMA were conjugated with fluorescent dye (EFL, China). Scaffolds were observed and 3D-constructed by laser scanning confocal microscopy. The hydrogels were subjected to compression analysis via a universal testing machine (HY-0230, Hengyi, China) at a speed of 10 mm min^−1^. The release of IL-8 and NGF was measured with ELISA kits (Abmart, China). Briefly, scaffold was immersed in 1 ml of PBS supplemented with 1% streptomycin and penicillin at 37 °C. At the designated time points, the supernatant was collected for IL-8 or NGF measurement.

### scRNA-seq and bulk RNA-seq data reanalysis based on GEO database

Transcriptomic data (GSE182923, GSE225224, GSE272612) were retrieved from the Gene Expression Omnibus (GEO) database and analyzed for enrichment in GO and KEGG. For scRNA-seq (GSE272612), cells were grouped using an unsupervised graph-based clustering approach. The resulting clusters were visualized using *t*-distributed stochastic neighbor embedding (t-SNE). Cluster-specific marker genes were identified with the FindAllMarkers function using the Wilcoxon rank-sum test. Cell type annotation was then performed based on canonical marker genes and validated with reference datasets.

### Configuration of diabetic CM

To mimic the hyperglycemic milieu of diabetes and culture cells isolated from diabetic mice, the in vitro diabetic CM (DCM) was prepared as previously described [102], by supplementing complete Dulbecco’s modified Eagle’s medium (containing 10% FBS and 1% penicillin/streptomycin) with 25 mM glucose and 500 μM bovine serum albumin–palmitate solution. This formulation can effectively recapitulate the diabetic microenvironment in vitro. All in vitro cell experiments were conducted using DCM as the basic culture medium.

### Isolation and culture of primary neutrophils and macrophages

The MojoSort Mouse Ly6-G Selection Kit (480123, BioLegend, USA) and the MojoSort Mouse F4/80 Selection Kit (480170, BioLegend, USA) were employed to isolate neutrophils and macrophages, respectively, from the femoral healing tissues of 14-week-old mice. Animals were humanely euthanized via cervical dislocation. Subsequently, bone healing tissues were harvested by flushing the femurs with heparinized culture medium using a 1-ml syringe, followed by fine mincing with ophthalmic scissors. The tissues were then incubated in an enzymatic cocktail—comprising deoxyribonuclease I (300 U/ml), collagenase type IV (500 U/ml), and hyaluronidase (2,700 U/ml; all from Biofroxx, Germany)—at 37 °C with shaking at 90 rpm for 1 h. After dissociation through a cell strainer and centrifugation (500*g*, 5 min), the cell pellet was resuspended and subjected to biotin-antibody cocktail and streptavidin nanobead treatment according to the manufacturer’s instructions. Magnetic separation was performed to specifically segregate Ly6G^+^ neutrophils or F4/80^+^ macrophages. Post-isolation, cells were maintained in either DCM for the diabetes group or complete medium for the control group. To induce neutrophil apoptosis in vitro, cells were treated with staurosporine (STS), a broad-spectrum protein kinase inhibitor that activates Bax-/Bak-dependent mitochondrial pathways [103].

### Neutrophil depletion

The methodology and validation of neutrophil depletion were performed as described in our previous study [38]. Intraperitoneal injection of rat anti-mouse Ly6G antibody (clone 1A8; Bio X Cell, USA) was performed to deplete neutrophils. An initial dose of 200 μg per mouse was administered 2 d before surgery, followed by 100 μg every other day until euthanasia. The control mice received an equivalent dose of rat immunoglobulin G (IgG).

### Cell migration assay using fibrin gel

The migration of neutrophils through a fibrin gel was evaluated following stimulation with extracts from different scaffolds. Briefly, CCC scaffolds containing varying concentrations of hyaluronidase were placed at the bottom of 24-well plates and incubated in complete medium for 24 or 120 h to obtain scaffold extracts. DIO-labeled neutrophils were centrifuged at 1,000 rpm for 5 min and resuspended in prewarmed fibrinogen solution (3 mg/ml). Fibrin gels were prepared by mixing 25 μl of thrombin (1 mg/ml) with 475 μl of the neutrophil–fibrinogen suspension (1 × 10^4^ cells per well) and casting the mixture into 24-well plates. After gelation, 500 μl of scaffold extract was added atop the gel. At 24 h post-seeding, the extract was removed, and the gels were fixed with 2.5% glutaraldehyde at 4 °C for 15 min.

Cell distribution in the gels was imaged using laser scanning confocal microscopy (Sp8, Leica, Germany). 3D images were reconstructed using LAS X (Leica, Germany). For each group, 3 *x*-*z* sections of the reconstructed 3D images were randomly chosen, average distance from upper edge of cell distribution to the top of the gel was measured by ImageJ software, the data were quantified as average migration distance of the cells.

### Isolation and culture of DRG neurons and siRNA transfection

As previously validated protocol [104], DRGs were isolated from diabetic mice following humane euthanasia, after which the L3–L5 spinal segments were carefully harvested. The DRGs were then precisely removed from the dorsal spine area and placed immediately into ice-cold DCM. After washing in fresh medium, the ganglia were subjected to 30 min of digestion at 37 °C in 0.05% trypsin–EDTA. The digested cells were seeded on poly-l-lysine-coated 6-well plates filled with DCM, and DRGs from individual mice were assigned to each well. The following day, the medium was refreshed and enriched with 10 μM cytosine arabinoside (ARA-C) to inhibit non-neuronal cell proliferation, with subsequent medium changes occurring every other day. Gal-3 siRNAs were purchased from Invitrogen, and transfection was performed with Lipofectamine RNAiMAX reagent (Invitrogen) following the manufacturer’s instructions. The sequences of mouse Gal-3 siRNAs were 5′-AUGAUUGUGAUCAGCAUGCTT-3′ [105].

### Bulk RNA sequencing

To instigate transcriptomic alterations after CM stimulation, the macrophages were harvested and preserved at −20 °C. The extraction of RNA was performed using TRIzol (Invitrogen, USA), and the integrity of the RNA was assessed via agarose gel electrophoresis, a NanoPhotometer (Implen, Germany), and an Agilent 2100 bioanalyzer (Agilent Technologies, USA). Following mRNA enrichment and fragmentation, library construction was performed using NEB Buffer (New England Biolabs, USA) and the TruSeq Kit (Illumina, USA). These libraries were quantified and sequenced on an Illumina HiSeq2000 platform using the TruSeq SBS Kit v3-HS. The sequencing data were subjected to quality control with FastQC, adapter sequences were trimmed using Trimmomatic, HISAT2 was used for reads alignment, and HTSeq was used for quantifying gene expression. DESeq2 was used for differential expression analysis, and DAVID was used for enrichment analysis. Data visualization was facilitated through R and Bioconductor packages.

### Scanning electron microscopy

For SEM observation, samples were prepared and imaged using a scanning electron microscope (Sigma 360, ZEISS, Germany). Prior to imaging, the specimens were dehydrated and coated following a standard preparation protocol. Briefly, DRG neurons and macrophages cocultured with different scaffolds were harvested. The samples were immediately fixed in 2.5% glutaraldehyde (Sinopharm Chemical Reagent Co. Ltd., China) at 4 °C overnight. After fixation, samples were rinsed 3 times with PBS (10×, pH 7.4; Servicebio, China) for 15 min each. Post-fixation was performed with 1% osmium tetroxide (OsO₄; Ted Pella Inc., USA) for 1 h, followed by 3 additional PBS rinses. Samples were then sequentially dehydrated in graded ethanol solutions (30%, 50%, 70%, 90%, and 100%; Sinopharm Chemical Reagent Co. Ltd., China) for 15 to 20 min each step. The dehydrated specimens were dried using a critical point dryer (EM CPD300, LEICA, Germany) and subsequently sputter-coated with platinum using an ion sputtering coater (Mini Coater, SuPro Instruments, China) for 70 s. Finally, the surface morphology of DRG neurons and macrophages was examined under the SEM at various magnifications.

### Transmission electron microscopy

Ultrathin sections were prepared from cocultured cells embedded in Matrigel. Fresh cells were fixed in 2.5% glutaraldehyde at 4 °C for 24 h. After fixation, the cells were rinsed 3 times with PBS (pH 7.4) for 15 min each. The samples were then post-fixed in 1% OsO₄ for 2 h, followed by 3 additional rinses in 0.1 M PBS (pH 7.4) for 15 min each. Dehydration was performed through a graded ethanol series, and the samples were infiltrated with epoxy embedding medium. Ultrathin sections (70 nm) were subsequently cut, mounted, and stained. The sections were examined and imaged using a HITACHI HT7800 transmission electron microscope operated at 80 kV.

### In vitro evaluation of innervated osteogenesis potential

ALP activity, mineralization staining, immunofluorescent staining, and RT-PCR were performed to evaluate the ability of CCC scaffolds to promote innervated osteogenesis in BMSC isolated from diabetic mice. In this experiment, a coculture model comprising DRG neurons, BMSC, and scaffolds was established. Scaffolds from different groups were placed in the upper chamber of a transwell system, while DRG neurons were seeded in the lower chamber. After 72 h of coculture, the medium was collected and centrifuged at 1,000 rpm for 5 min to remove any remaining cells and frozen at −80 °C for future use. Additionally, collected medium was filtered using a 0.22-μm filter and mixed with fresh DCM at a ratio of 1:2 as CM for subsequent BMSC culture.

MSCs were seeded in 24-well plates at a density of 3 × 10^4^ cells per well. After 24 h, the medium was replaced with the CM. After 7 d of culture, ALP staining and activity assay (Beyotime, China) were performed. In addition, 2 osteogenic- related proteins (RUNX2 and OCN) were evaluated by immunofluorescence staining. Total RNA was extracted from the treated MSCs to quantify the expression of osteogenesis-related genes (Bglap, Spp1, and Col1a1) by RT-qPCR. After culturing for 14 d, the extracellular matrix (ECM) mineralization was assessed by the Alizarin Red Staining (Beyotime, China).

### In vitro assessment of Gal-3 production and neurite outgrowth of DRG neurons

To assess the Gal-3 level of DRG neurons stimulated by various groups of scaffolds, an indirectly coculture model of DRG neurons and scaffolds was established: DRG neurons were seeded in lower chamber of transwell system, and scaffolds were placed in upper chamber. The expression of Gal-3 was measured with immunofluorescent staining and ELISA kits (Abmart, China). Briefly, scaffolds and neurons were cocultured in 1 ml of DCM at 37 °C. At the designated time points, 10 μl of supernatant was collected for Gal-3 measurement. After 120 h of culture, DRG neurons were fixed with 4% paraformaldehyde and subjected to immunofluorescent staining for Gal-3 and the neuronal fiber marker Tuj-1. Images were acquired using a laser-scanning confocal microscope (SP8, Leica, Germany), and neurite length was quantified using ImageJ software with the NeuronJ plugin. In addition, total RNA was extracted from DRG neurons after 120 h of culture, and the expression levels of Gal-3, Tuj-1, and the sensory neuropeptides CGRP and SP were analyzed by RT-PCR.

### In vitro validation of macrophage efferocytosis of neutrophils

To evaluate the efferocytosis of macrophages on apoptotic neutrophils, primary neutrophils isolated from the bone marrow of mice were used as model cells. First, neutrophil apoptosis was induced by STS (0.5 μm for 3 h), and apoptotic cells were labeled with DIO cell membrane dye (Yeasen, China). Subsequently, these neutrophils were added to macrophages expressing red fluorescent protein at a ratio of 5:1 (neutrophils to macrophages) in the lower chamber of a transwell system containing DCM. Scaffolds from different groups were placed in the upper chamber to provide stimulation during the 24-h coculture period. The remaining cells were observed through a laser- scanning confocal microscopy (Sp8, Leica, Germany). Additionally, the macrophage efferocytosis capacity was evaluated through the expression of CD36 assessed by RT-PCR.

### Construction of critical mandibular bone defect model

Critical bone defect surgery was performed on mandible in adult mice/rats as adapted and modified from previously validated protocol [85,106]. Animals were anesthetized with isoflurane gas for the duration of the experiment. The skin was carefully shaved and then disinfected with alcohol. A sterile surgical blade was utilized to make an incision in the skin. The incision extended through the entire thickness of the muscle around the mandibular angle and the underlying periosteum. Subsequently, the skin and periosteum were excised to expose the mandibular bone. After identifying anatomical landmarks, experimental critical mandibular bone defects were created on both sides using a bur (4.0-mm-diameter bone trephine bur for rats; 2.0-mm-diameter round bur for mice) mounted on a rotary handpiece and irrigated with 0.9% saline solution. Then, scaffolds from various groups were placed in defects. Following the surgical procedure, the muscle and skin layers were repositioned and sutured closed using 5-0 resorbable sutures. All animals were monitored until they recovered from anesthesia, at which point they were returned to their cages. A soft diet was provided for the first 7 d postoperatively, followed by a regular diet until euthanization.

### Flow cytometry

Following euthanasia, the defect segments of mouse mandible were promptly immersed in ice-cold PBS. The tissues were finely minced using ophthalmic scissors and incubated in an enzymatic cocktail (deoxyribonuclease I at 300 U/ml, collagenase type IV at 500 U/ml, and hyaluronidase at 2,700 U/ml; all from Biofroxx, Germany) at 37 °C with shaking at 90 rpm for 1 h. After incubation, the tissues were further dissociated through a cell strainer, and the filtrate was centrifuged at 500*g* for 5 min to collect the cells. The cells were then resuspended in PBS to obtain single-cell suspensions for immunofluorescence staining. The following reagents and antibodies were used for immunofluorescence staining: Fixable Viability Stain 700 (564997, BD,USA), purified rat anti-CD16/CD32 mouse Fc Blocker (553141, BD), BV510 rat anti-CD45 (563891, BD), PE hamster anti-CD29 (562801, BD), PerCP-Cy5.5 rat anti-CD44 (560570, BD), BV 421 rat anti-Ly-6G (127627, BioLegend, USA), fluorescein isothiocyanate (FITC) rat anti-CD11b (101205, BioLegend), PE-Cy7 rat anti-CD86 (105013, BioLegend), and AF647 rat anti-CD206 (141711, BioLegend). Specific populations, including neutrophils (CD45^+^Ly6G^+^), mature neutrophils (CD45^+^Ly6G^+^CD11b^+^), N2 neutrophils (CD45^+^Ly6G^+^CD206^+^), and mesenchymal stem cells (CD45^−^CD29^+^CD44^+^), were identified and quantified. Analysis was conducted using a FACS Fortessa cytometer (BD Biosciences) and FlowJo software.

### RNA isolation and qPCR

Following euthanasia, cells or bone healing tissue isolated from mice or rats was immediately homogenized in RNAiso Plus reagent (T9108, TaKaRa, Japan) for total RNA extraction. The extracted RNA was then reverse transcribed into cDNA using the PrimeScript RT Reagent Kit (RR036A, TaKaRa, Japan). qRT-PCR analyses were conducted on a LightCycler 480 II system (Roche, Switzerland) utilizing iQ SYBR Green Supermix (Bio-Rad). Gapdh served as the internal control for normalization. The RT-PCR primers used were as follows: mouse *Bglap*, 5′-CGCAATAAGGTAGTGAACAGACTCC-3′ and 5′-CCATAGATGCGTTTGTAGGCGG-3′; mouse *Spp1,* 5′-GCTTGGCTTATGGACTGAGGTC-3′ and 5′-CCTTAGACTCACCGCTCTTCATG-3′; mouse *col1a1*, 5′-CCTCAGGGTATTGCTGGACAAC-3′ and 5′-CAGAAGGACCTTGTTTGCCAGG-3′; mouse *Gal-3* (*Lgals3*), 5′-AACACGAAGCAGGACAATAACTGG-3′ and 5′-GCAGTAGGTGAGCATCGTTGAC-3′; mouse *Tuj-1* (*Tubb3*), 5′-CATGGACAGTGTCGGTCTG-3′ and 5′-TTCCGCACGACAGTTTCATC-3′; mouse Gas-6, 5′-CGGCATTCCCTTCAAGGAGAGT-3′ and 5′-CTCAACTGCCAGGACCACCAACT-3′; mouse Pros1, 5′-GCACAGTGCCCTTTGCCT-3′ and 5′-CAAATACCACAATATCCTGAGACGTT-3′; mouse Mfge8, 5′-GAGCAACAGTGCCAAGGAATGG-3′ and 5′-ACTGTGGGCTACCTTGTAGGAC-3′; mouse Tulp1, 5′-CAGGAAACGCAAGCGGAGTAAG-3′ and 5′-TTCTGCCCGTTGTCAAAGACCG-3′; mouse *CD36*, 5′-GGACATTGAGATTCTTTTCCTCTG-3′ and 5′-GCAAAGGCATTGGCTGGAAGAAC-3′; *Il-1b*, 5′-TGGACCTTCCAGGATGAGGACA-3′ and 5′-GTTCATCTCGGAGCCTGTAGTG-3′; mouse *Tnfα*, 5′-CCCTCACACTCAGATCATCTTCT-3′ and 5′-GCTACGACGTGGGCTACAG-3′; *Mpo*, 5′-CGTGTCAAGTGGCTGTGCCTAT-3′ and 5′-AACCAGCGTACAAAGGCACGGT-3′; *Il-10*, 5′-CGGGAAGACAATAACTGCACCC-3′ and 5′-CGGTTAGCAGTATGTTGTCCAGC-3′; mouse *MMR* (*Mrc1*), 5′-GTTCACCTGGAGTGATGGTTCTC-3′ and 5′-AGGACATGCCAGGGTCACCTTT-3′; mouse *Actb*, 5′-CAGGTCGGTGTGAACGGATTTG-3′ and 5′-TGTAGACCATGTAGTTGAGGTCA-3′; rat *Vegf*, 5′-GGGCTGCTGCAATGATGAAG-3′ and 5′-GCTGGCTTTGGTGAGGTTTG-3′.

### ELISA

Samples or supernatant was initially immersed in PBS supplemented with protease inhibitor (Yeasen, China) and subjected to mechanical disruption with ophthalmic scissors, followed by homogenization using a tissue lyser (F6/10, Jingxin, China) and ultrasonication (VCX130, Sonics, USA) to ensure thorough cell lysis and protein solubilization. After homogenization, the samples were centrifuged at 12,000 rpm for 15 min in a refrigerated centrifuge to separate the supernatant for analysis. ELISAs were employed for the quantification of NGF (EK5203, Abmart), IL-8 (EK0453, Signalway), and Gal-3 (EK10101, Signalway). These assays were conducted according to the manufacturers’ protocols. Absorbance readings were obtained using a multifunctional microplate reader (Spark, Tecan, Switzerland), and concentrations were deduced from standard curves, ensuring the methodology’s conciseness and adherence to professional standards.

### Histology and IHC/ICC staining

Fixed femurs were decalcified in 20% EDTA for up to 1 month. Then, samples were processed for paraffin embedment (10-μm-thick sections). Longitudinal sections were mounted on adhesive slides. H&E staining and Masson staining were performed using standard protocols. For immunohistochemistry/immunocytochemistry (IHC/ICC) analysis, samples were washed 3 times in PBS for 5 min. Then, the sections or cells were first permeabilized with 0.5% Triton-X for 30 min. The cells, or the sections following antigen retrieval in citrate buffer (pH 6.0) with heat treatment, were blocked using a solution of 5% donkey serum and incubated with primary antibodies overnight at 4 °C. The following day, samples were washed in PBS, incubated in the appropriate fluorescent secondary antibody, and then mounted with antifade mounting medium with 4′,6-diamidino-2-phenylindole (DAPI). Digital images of these sections were captured with 10× to 40× objectives using confocal microscopy (Sp8, Leica, Germany).

For staining with primary antibodies from the same species, the Multicolor Fluorescent Immunohistochemical Staining Kit (Absin, abs50012) was used. Briefly, the samples were subjected to microwave-assisted antigen retrieval in 1× solution, followed by endogenous peroxidase quenching with 3% H_2_O_2_. After being washed with tris-buffered saline with Tween 20 (TBST), the samples were blocked and incubated with primary antibodies and then with horseradish peroxidase (HRP)-conjugated secondary antibodies. Amplification of fluorescent signals was achieved using tyramide signal amplification (TSA) fluorescent dyes, and after microwave treatment to remove bound antibodies, steps were repeated for different primary antibodies. The samples were washed and stained with DAPI. Samples were mounted with antifade medium and imaged using fluorescence microscopy (Sp8, Leica, Germany). The semiquantitative analysis was processed using ImageJ software and the NeuronJ plugin. The following antibodies were used for immunostaining: rat anti-Ly6G (Novus, NBP2-00441, 1:200 dilution), mouse anti-CD68 (Abcam, ab955, 1:3,000 dilution), mouse anti-Gal-3 (Proteintech, 60207, 1:200 dilution), mouse anti-Tuj-1 (CST, 45058, 1:200 dilution), and mouse anti-PGP9.5 (Abcam, ab8189, 1:50 dilution).

### Statistical analysis

All data were tested for normality by the Kolmogorov–Smirnoff test. Unpaired 2-tailed Student’s *t* test (parametric data) was used for simple comparisons. For comparisons of more than 2 groups, data were analyzed by one-way analysis of variance (ANOVA) test followed by Tukey’s post hoc tests or 2-way ANOVA test. Numerical data are graphed in bar charts with mean ± SEM. The significance level was set at *P* < 0.05; ns indicates no statistically significant difference; *P < 0.05, **P < 0.01, ***P < 0.001, ****P < 0.0001.

## Data Availability

All data are available in the main text or the Supplementary Materials.
