# Supplementary material for: Directing Neutrophil Fate via Sensory–Immune Interactions Accelerates Diabetic Bone Healing
Source: Research (Wash D C). 2026 Jun 24;9:1320. doi: 10.34133/research.1320 (PMC13291548; doi:10.34133/research.1320)
Supplement: Supplementary 1 — Figs. S1 to S8 [file research.1320.f1.docx]

SUPPLEMENTARY MATERIALS

Extended data Fig. 1 to 8


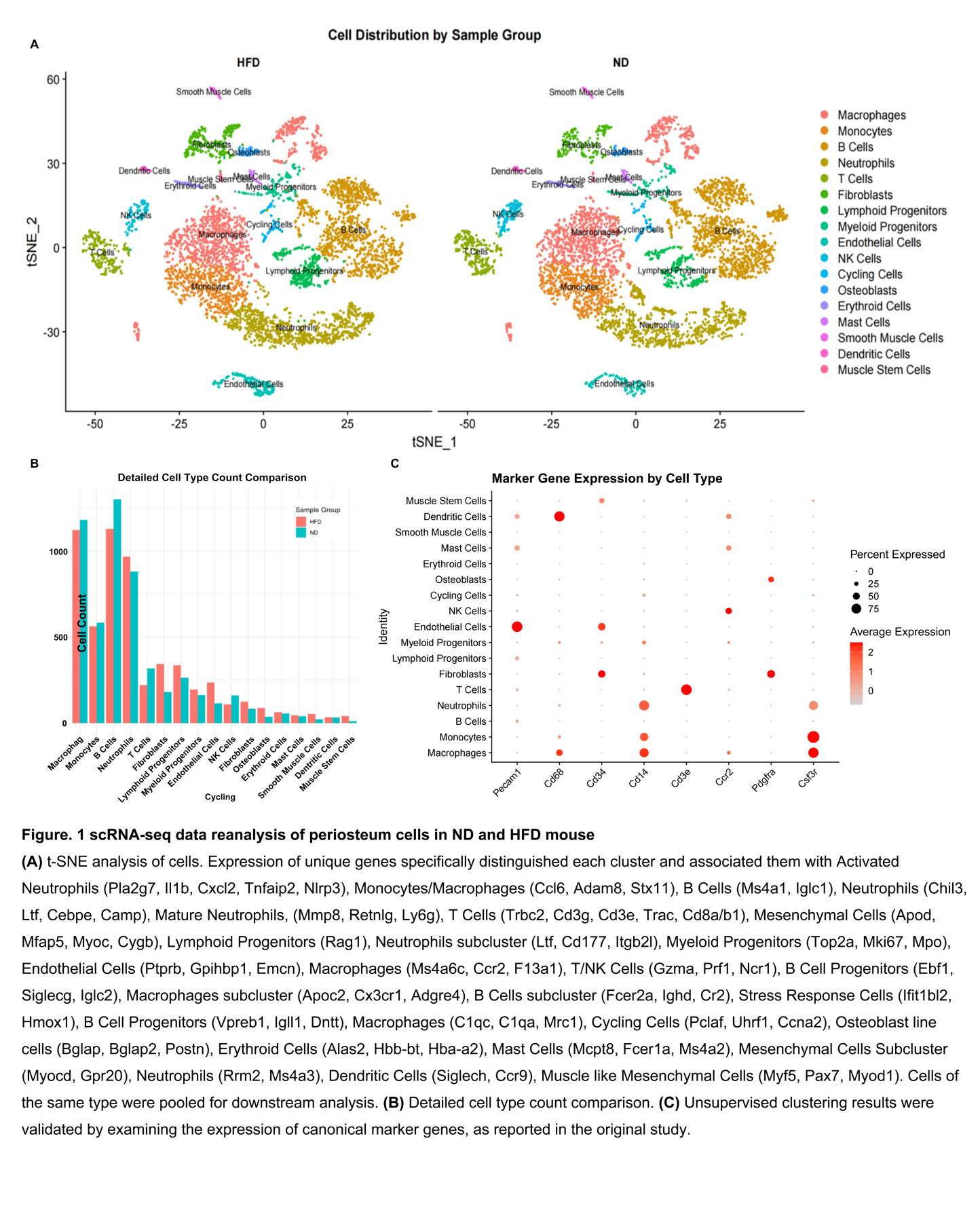


**Extended data Fig. 1 scRNA-seq data reanalysis of periosteum cells in ND and HFD mouse**

**(A)** t-SNE analysis of cells. Expression of unique genes specifically distinguished each cluster and associated them with Activated Neutrophils (Pla2g7, Il1b, Cxcl2, Tnfaip2, Nlrp3), Monocytes/Macrophages (Ccl6, Adam8, Stx11), B Cells (Ms4a1, Iglc1), Neutrophils (Chil3, Ltf, Cebpe, Camp), Mature Neutrophils, (Mmp8, Retnlg, Ly6g), T Cells (Trbc2, Cd3g, Cd3e, Trac, Cd8a/b1), Mesenchymal Cells (Apod, Mfap5, Myoc, Cygb), Lymphoid Progenitors (Rag1), Neutrophils subcluster (Ltf, Cd177, Itgb2l), Myeloid Progenitors (Top2a, Mki67, Mpo), Endothelial Cells (Ptprb, Gpihbp1, Emcn), Macrophages (Ms4a6c, Ccr2, F13a1), T/NK Cells (Gzma, Prf1, Ncr1), B Cell Progenitors (Ebf1, Siglecg, Iglc2), Macrophages subcluster (Apoc2, Cx3cr1, Adgre4), B Cells subcluster (Fcer2a, Ighd, Cr2), Stress Response Cells (Ifit1bl2, Hmox1), B Cell Progenitors (Vpreb1, Igll1, Dntt), Macrophages (C1qc, C1qa, Mrc1), Cycling Cells (Pclaf, Uhrf1, Ccna2), Osteoblast line cells (Bglap, Bglap2, Postn), Erythroid Cells (Alas2, Hbb-bt, Hba-a2), Mast Cells (Mcpt8, Fcer1a, Ms4a2), Mesenchymal Cells Subcluster (Myocd, Gpr20), Neutrophils (Rrm2, Ms4a3), Dendritic Cells (Siglech, Ccr9), Muscle like Mesenchymal Cells (Myf5, Pax7, Myod1). Cells of the same type were pooled for downstream analysis. **(B)** Detailed cell type count comparison. **(C)** Unsupervised clustering results were validated by examining the expression of canonical marker genes, as reported in the original study.

**
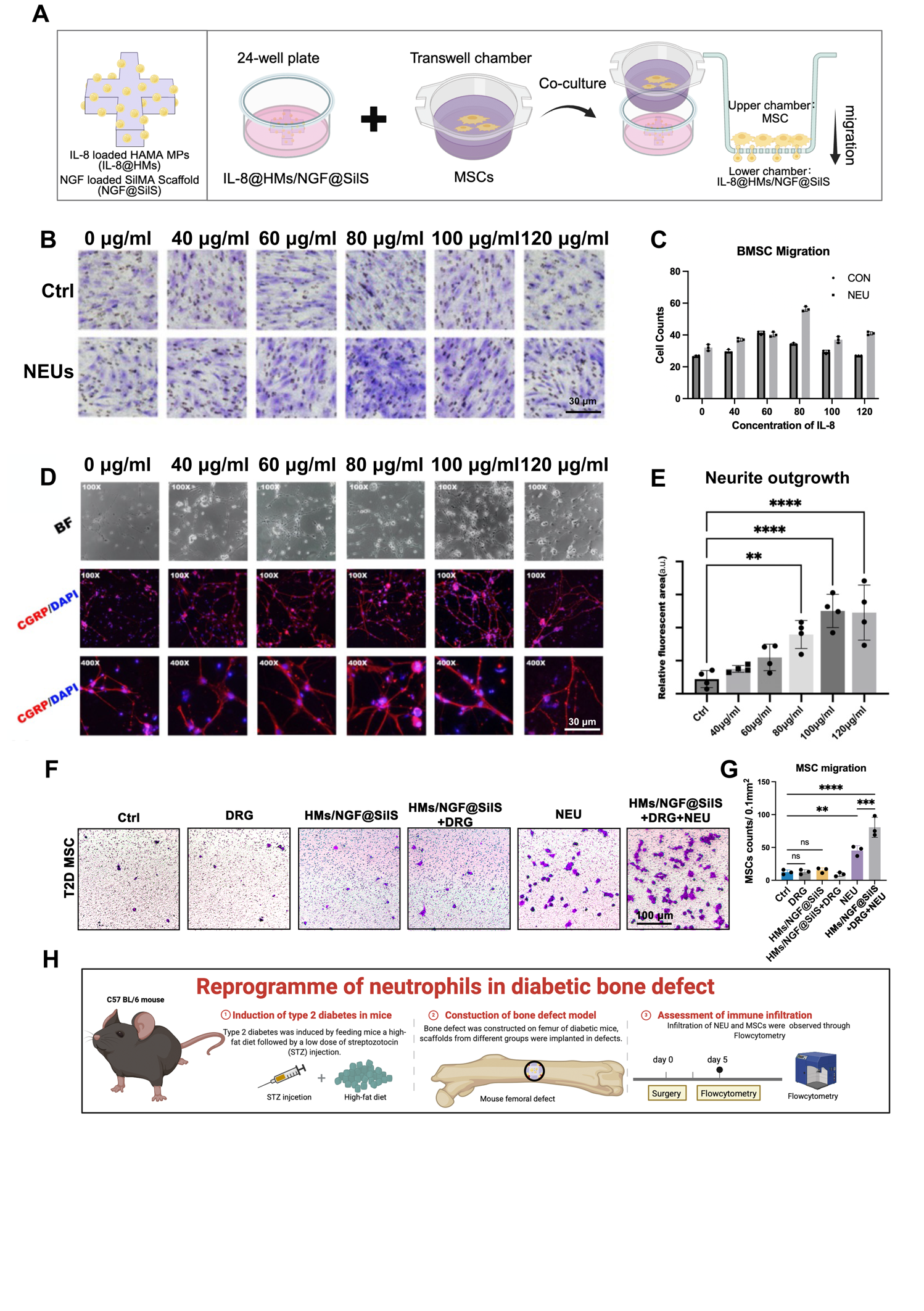
**

**Extended data Fig. 2 Optimization of IL-8 and NGF Concentrations.**

**(A)** Schematic illustration **(B)** Representative images and **(C)** Semi-quantitive evaluation of the Transwell assay assessing migration of BMSC co-cultured with scaffolds loading various concentration of IL-8, with/without neutrophils, Scale bars: 30 μm. **(D)** Representative images and **(E)** semi-quantative evaluation of bright-field and immunofluorescence staining for the sensory nerve marker CGRP, Scale bars: 30 μm. **(F)** Representative images and **(G)** Semi-quantitive evaluation of the Transwell assay assessing migration of T2D BMSC cultured in indicated groups, Scale bars: 200 μm. **(H)** Schematic illustration of the femoral defect model in T2D mice. Data are mean ± s.e.m. analyzed by one-way ANOVA and post-hoc multiple comparisons, ns indicates no statistically significant difference, *P < 0.05, **P < 0.01, ***P < 0.001, ****P < 0.0001.


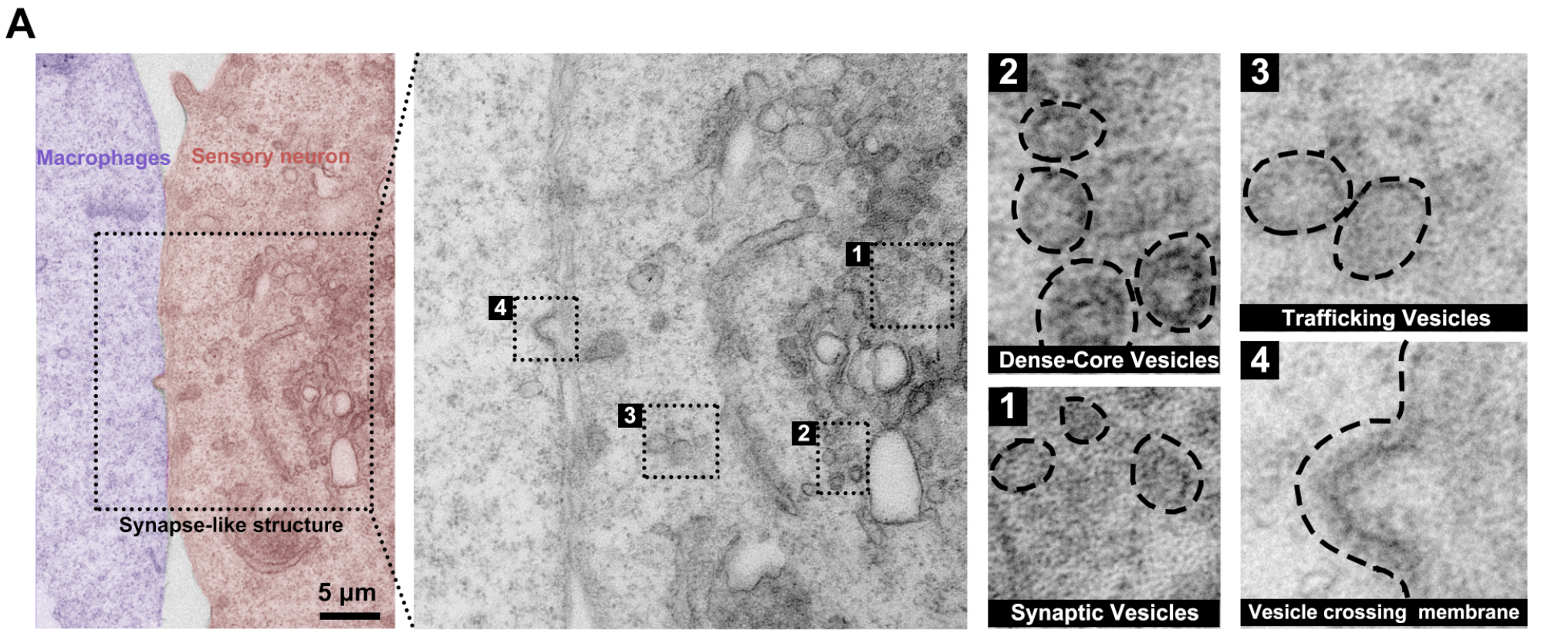


**Extended data Fig. 3 TEM analysis of neuron-macrophage contact area. (A)** Transmission electron microscopy (TEM) of DRG and macrophages showing the **(1)**synaptic vesicles, **(2)**dense-core vesicles, **(3)**trafficking vesicles and **(4)**vesicle crossing membrane.


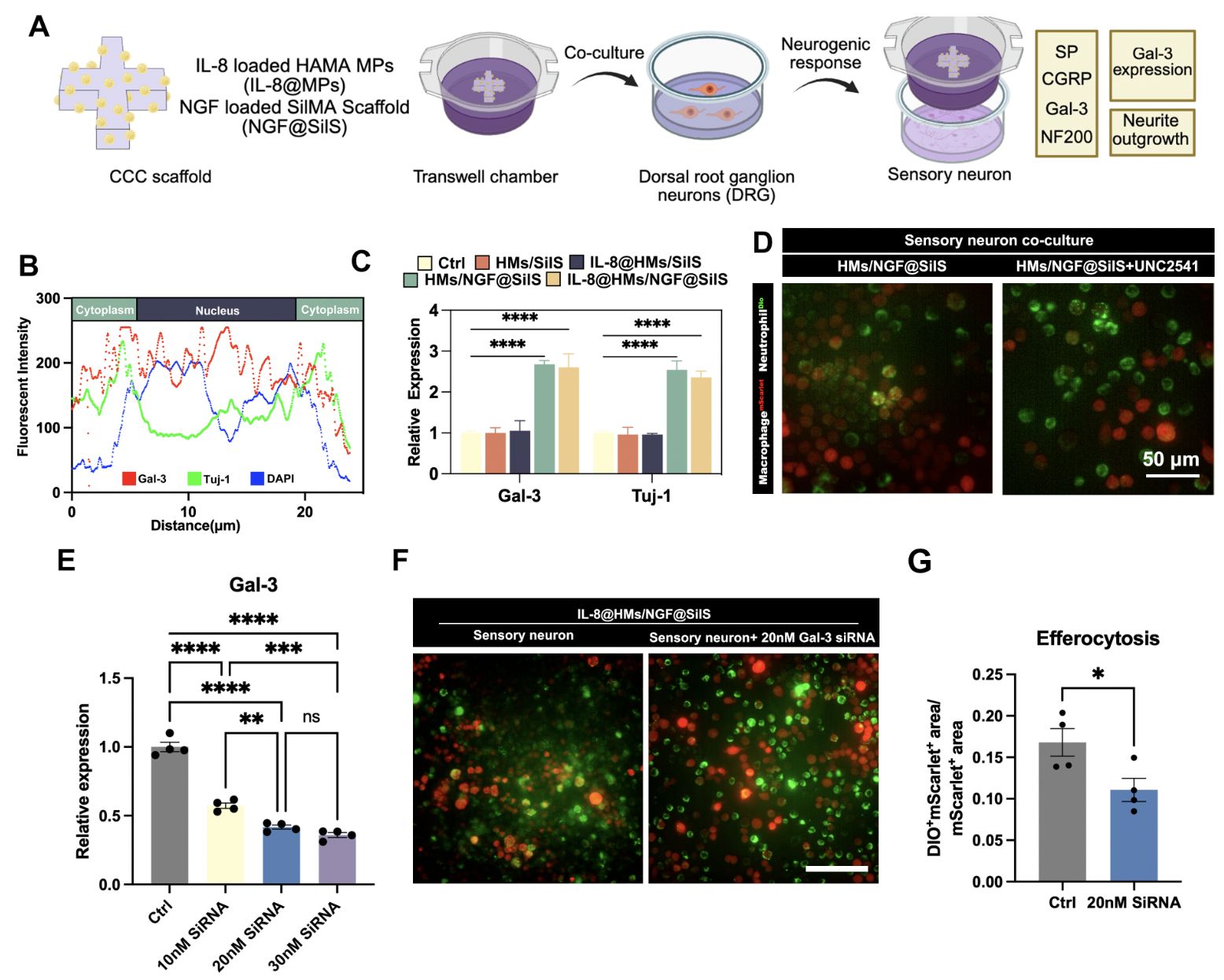


**Extended data Fig. 4 Evaluation of the neuronal Gal-3 and Gal-3-MerTK axis mediated efferocytosis.**

**(A)** Experimental design schematic. **(B)** Immunofluorescent co-localization analysis of Tuj-1 and Gal-3 in DRG neuron. **(C)** Gene expression of Gal-3 and Tuj-1was assessed in indicated groups (n=3). **(D)** Representative images of efferocytosis observed in co-culture of mScarlet-labeled macrophages and DIO-labeled neutrophils, following 72 h incubation in indicated groups. Scale bars: 50 μm. **(E)** mRNA levels of galectin-3 after transfection of DRG neurons with different concentrations (10, 20, 30 nmol/L) of scrambled siRNA and galectin-3 siRNA. Total RNA was obtained after transfection for 48 h, and cells were harvested and analyzed by real time-PCR. **(F)** Representative images and **(G)** semi-quantitative assessment of efferocytosis observed in co-culture of mScarlet-labeled macrophages and DIO-labeled mouse neutrophils, following 72 h co-culture with DRG pre-treated with indicated condition.Scale bar: 100 μm. Data are mean ± s.e.m. analyzed by one-way ANOVA and post-hoc multiple comparisons, ns indicates no statistically significant difference, *P < 0.05, **P < 0.01, ***P < 0.001, ****P < 0.0001.


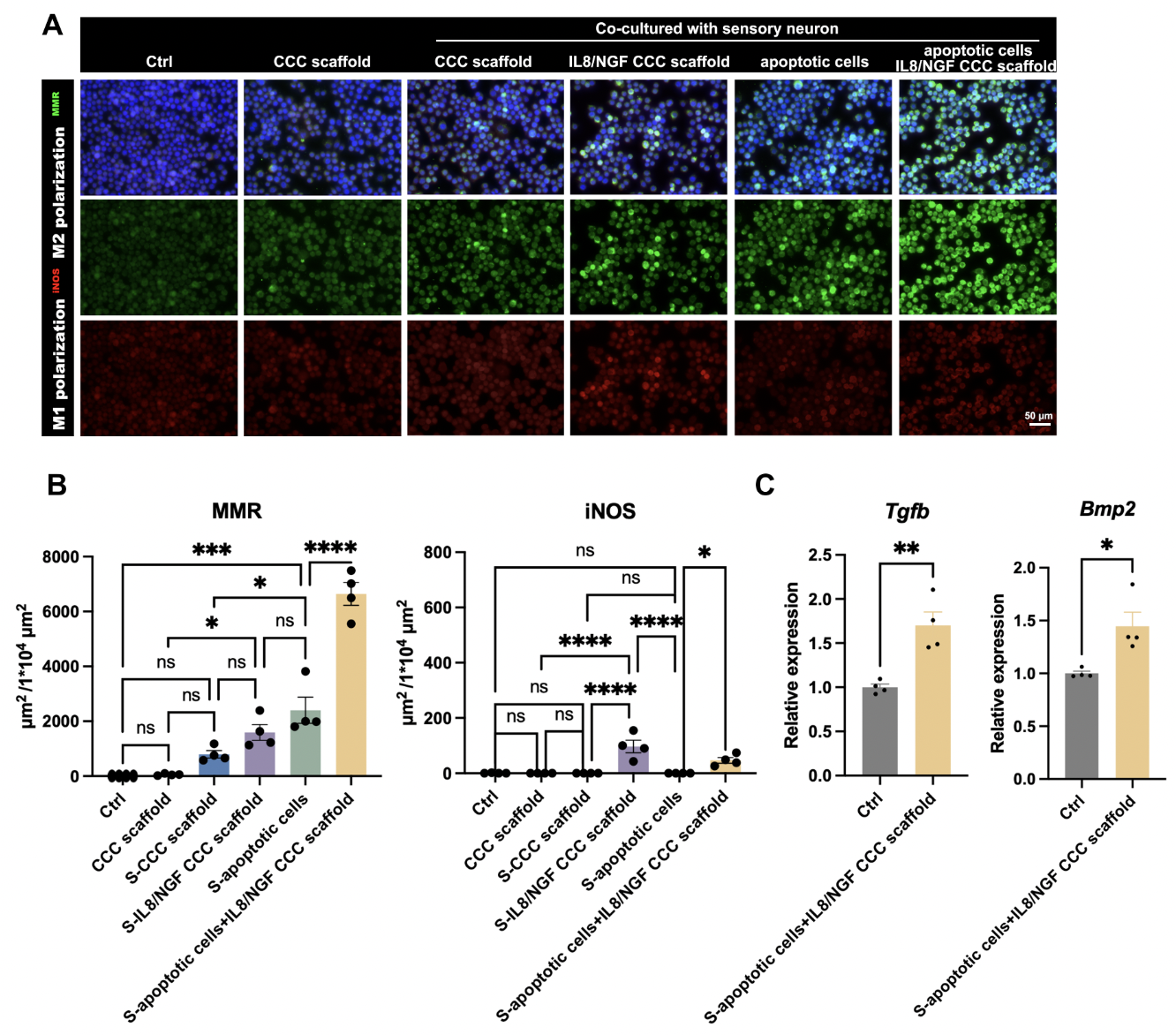


**Extended data Fig. 5 Polarization of macrophages stimulated by scaffolds and apoptotic cells.**

**(A)** Representative images, **(B)** semi-quantitative immunofluorescent analysis and **(C)** q-PCR analysis of macrophages cultured under indicated condition for 5 days. S- represents co-culture with sensory neuron. Scale bars represent 500 μm and 100 μm.


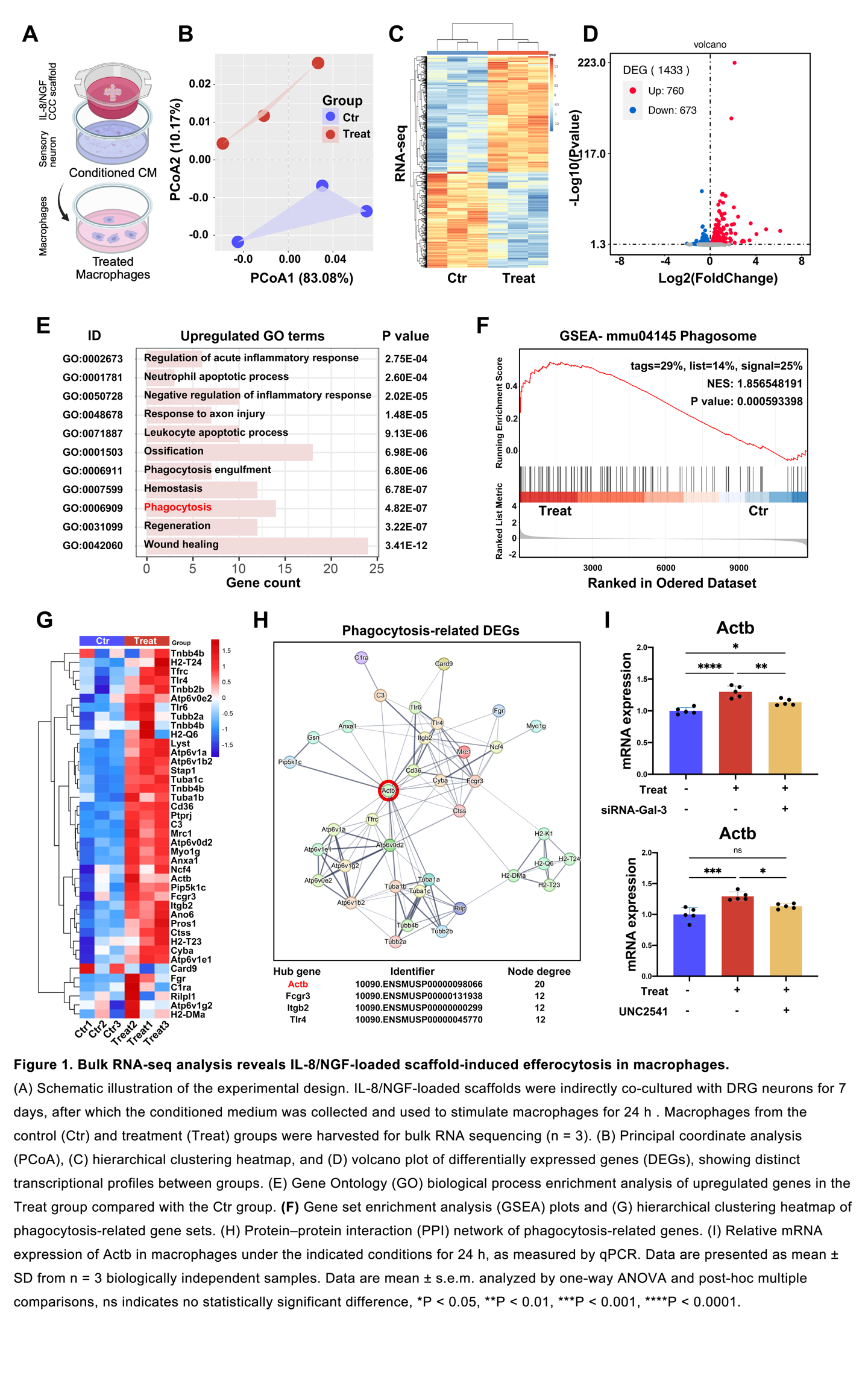


**Extended data Fig. 6 Bulk RNA-seq analysis reveals IL-8/NGF-loaded scaffold-induced efferocytosis in macrophages.**

**(A)** Schematic illustration of the experimental design. IL-8/NGF-loaded scaffolds were indirectly co-cultured with DRG neurons for 7 days, after which the conditioned medium was collected and used to stimulate macrophages for 24 h. Macrophages from the control (Ctr) and treatment (Treat) groups were harvested for bulk RNA sequencing (n = 3). **(B)** Principal coordinate analysis (PCoA), **(C)** hierarchical clustering heatmap, and **(D)** volcano plot of differentially expressed genes (DEGs), showing distinct transcriptional profiles between groups. **(E)** Gene Ontology (GO) biological process enrichment analysis of upregulated genes in the Treat group compared with the Ctr group. **(F)** Gene set enrichment analysis (GSEA) plots and **(G)** hierarchical clustering heatmap of phagocytosis-related gene sets. **(H)** Protein–protein interaction (PPI) network of phagocytosis-related genes. **(I)** Relative mRNA expression of *Actb* in macrophages under the indicated conditions for 24 h, as measured by qPCR. Data are mean ± s.e.m. analyzed by one-way ANOVA and post-hoc multiple comparisons, ns indicates no statistically significant difference, *P < 0.05, **P < 0.01, ***P < 0.001, ****P < 0.0001.


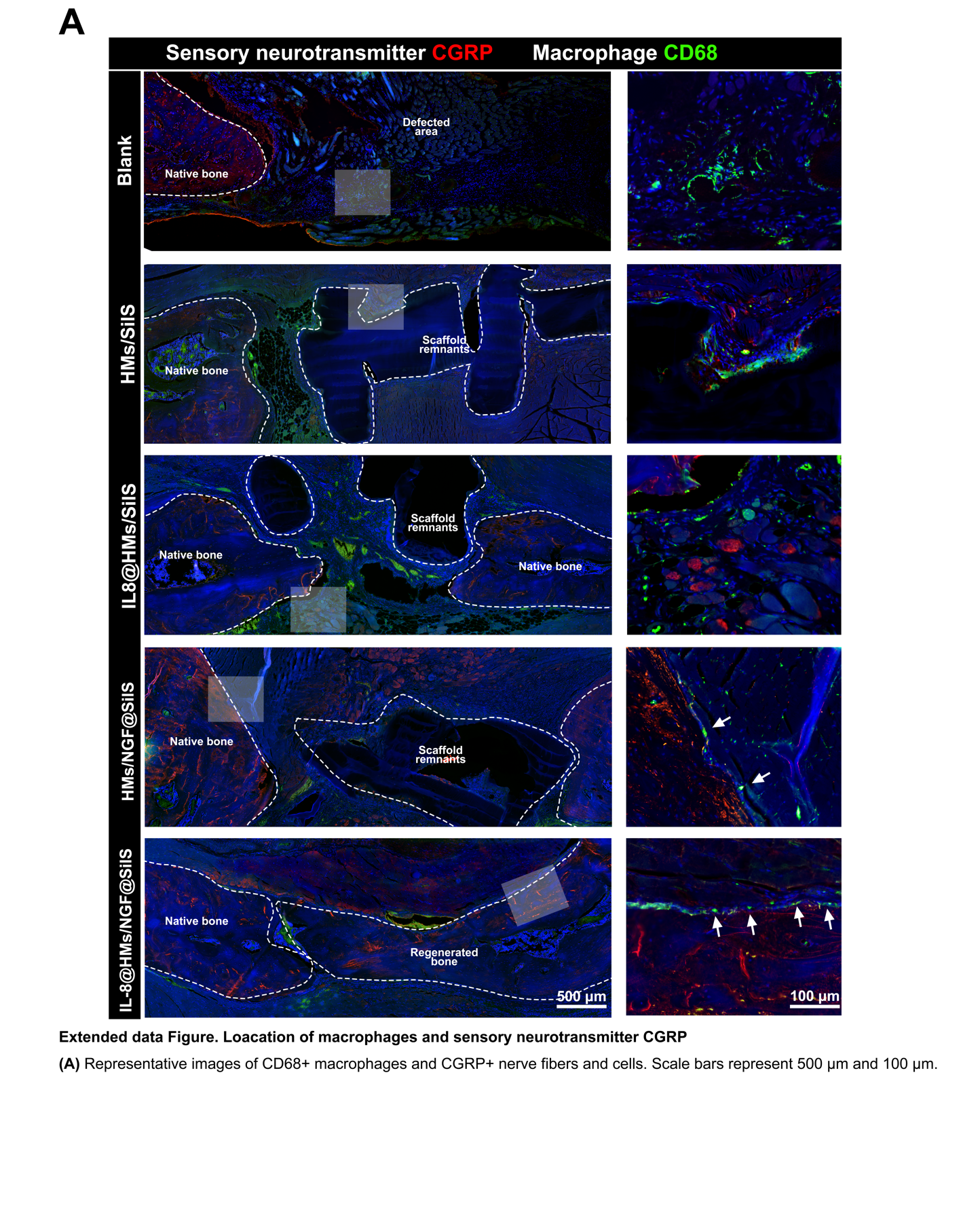


**Extended data Figure. 7 Immunostaining of macrophages and sensory neurotransmitter CGRP**

**(A)** Representative images of CD68+ macrophages and CGRP+ nerve fibers and cells. Scale bars represent 500 μm and 100 μm.


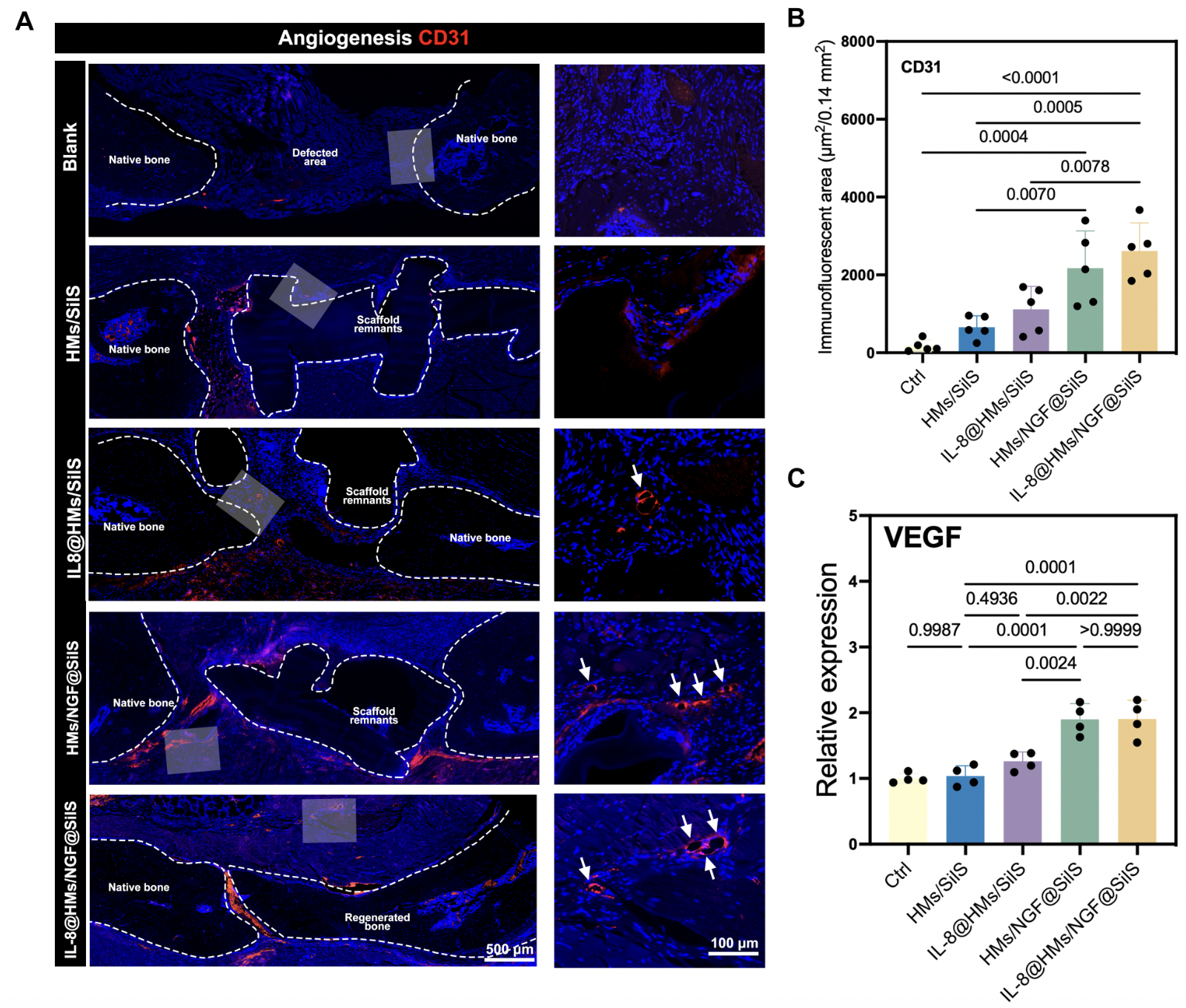


**Extendeda data Fig. 8 NGF-loaded CCC scaffolds promote angiogenesis.**

**(A)** Representative images and **(B)** semi-quantitative analysis of CD31+ vessels. Scale bars represent 500 μm and 100 μm.**(C)** Relative expression of *Vegf* in regenerating rat mandible on 7 days post implantation.
